# Synthesis and biological activity of myricetin derivatives containing 1,3,4-thiadiazole scaffold

**DOI:** 10.1186/s13065-017-0336-7

**Published:** 2017-10-17

**Authors:** Xinmin Zhong, Xiaobin Wang, Lijuan Chen, Xianghui Ruan, Qin Li, Juping Zhang, Zhuo Chen, Wei Xue

**Affiliations:** 1 State Key Laboratory Breeding Base of Green Pesticide and Agricultural Bioengineering, Key Laboratory of Green Pesticide and Agricultural Bioengineering, Ministry of Education, Guizhou University, Guiyang, 550025 China; 2 Key Laboratory of Monitoring and Management of Crop Diseases and Pest Insects, Ministry of Agriculture, Nanjing Agricultural University, Nanjing, 210095 China

**Keywords:** Myricetin, 1,3,4-thiadiazole, Antibacterial activity, Antiviral activity

## Abstract

**Background:**

Myricetin and 1,3,4-thiadiazole derivatives were reported to exhibit favorable antiviral and antibacterial activities. Aiming to discover novel myricetin analogues with potent activities, a series of novel myricetin derivatives containing 1,3,4-thiadiazole moiety were synthesized, and their antibacterial and antiviral activities were evaluated.

**Result:**

Bioassay results indicated that some target compounds exhibited potential antibacterial and antiviral activities. Among them, compounds **2**, **3a**, **3b**, **3d**, **3f**, **3i, 3m** and **3p** exhibited excellent antibacterial activities against *Xanthomonas oryzae pv. Oryzae* (*Xoo*), with EC_50_ values of 42.7, 38.6, 20.8, 12.9, 22.7, 27.3, 18.3 and 29.4 μg/mL, respectively, which were better than that of *thiadiazole*-*copper* (94.9 μg/mL). Compounds **3b**, **3d**, **3e**, **3f**, **3i** and **3o** showed good antibacterial activities against *Ralstonia solanacearum* (*Rs*), with EC_50_ values of 37.9, 72.6, 43.6, 59.6, 60.6 and 39.6 μg/mL, respectively, which were superior to that of *thiadiazole*-*copper* (131.7 μg/mL). In addition, compounds **3d**, **3f**, **3i** and **3m** showed better curative activities against *tobacco mosaic virus* (TMV), with EC_50_ values of 152.8, 99.7, 127.1, and 167.3 μg/mL, respectively, which were better than that of *ningnanmycin* (211.1 μg/mL).

**Conclusions:**

A series of myricetin derivatives containing 1,3,4-thiadiazole scaffold were synthesized, and their antibacterial activities against *Xoo* and *Rs* and their antiviral activity against TMV were evaluated. Bioassays indicated that some target compounds exhibited potential antibacterial and antiviral activities. These results indicated this kind of myricetin analogues could be further studied as potential alternative templates in the search for novel antibacterial and antiviral agents.

**Electronic supplementary material:**

The online version of this article (doi:10.1186/s13065-017-0336-7) contains supplementary material, which is available to authorized users.

## Background

The rational use of agrochemicals plays a pivotal role in agricultural production by effectively controlling plant diseases [1, 2]. Unfortunately, the application of traditional pesticides is greatly limited due to their negative impacts on the environment and the rapid emergence of resistance [2, 3]. Therefore, searching for high-efficiency and environmentally friendly agrochemicals remains an arduous challenge in pesticide chemistry [1, 4]. In this process, natural products and their derivatives with new modes of action have been developed as pesticides that are safe to the environment [5, 6].

**Fig. 1:**
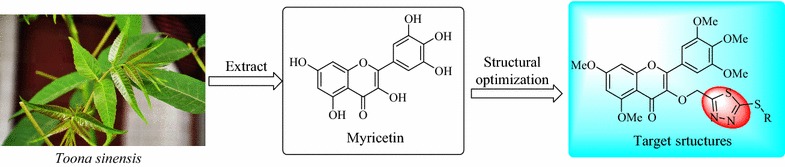
Design strategy for target molecules

As one of important natural products in medicinal chemistry, myricetin was reported to exhibit extensive bioactivities including antibacterial [7], antiviral [8], anticancer [9], anti-inflammatory [10], antioxidant [11], and hypoglycemic activities [12]. Our previous study extracted a mixture containing myricetin from the bark of *Toona sinensis* and found it to exhibit moderate antiviral activity against *tobacco mosaic virus* (TMV) [13]. Using natural myricetin as the lead molecule, some myricetin derivatives bearing Schiff-base moiety, which displayed good inhibitory activity against telomerase and excellent anticancer activity against human breast cancer cells MDA-MB-231, were synthesized by Xue et al. [14]. Furthermore, the acceptable antibacterial activities against *Xanthomonas oryzae pv. oryzae* (*Xoo*) and *Ralstonia solanacearum* (*Rs*) of myricetin derivatives containing acidamide moiety were also recently reported by us [15]. Obviously, myricetin derivatives as possible active ingredients play a key role in the searching for novel agrochemicals and pharmaceuticals (Fig. 1).

1,3,4-Thiadiazoles, which represent important nitrogenous heterocycles in medicinal chemistry, have attracted much attentions because of their various pharmacological activities, including antibacterial [16], antifungal [17], antiviral [18], anticonvulsant [19], anxiolytic [20], antinociceptive [21] and anticancer [22] activities. Among the above biological activities, acceptable antibacterial and antiviral activities displayed by 1,3,4-thiadiazoles have been reported well by chemists in recent years. For example, Li et al. [23] found that some 1,3,4-thiadiazole sulfone derivatives exhibited satisfactory antibacterial activities against rice bacterial leaf blight and leaf streak. Recently, we also found some 1,3,4-thiadiazole derivatives bearing 1,4-pentadiene-3-one moiety to exhibit remarkable antiviral activities against plant viruses [24].

**Scheme 1:**
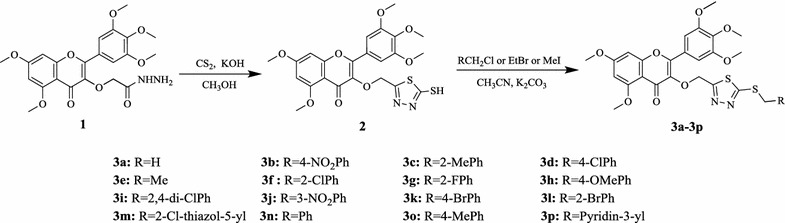
Synthetic route to the title compounds **3a**–**3p**

Considering these above results, we speculated that introducing 1,3,4-thiadiazole fragment into myricetin might generate novel lead compounds with greater biological activities. Thus, a series of myricetin derivatives containing 1,3,4-thiadiazole scaffold were synthesized (Scheme 1), and their antibacterial activities against *Xoo* and *Rs* and their antiviral activity against TMV were evaluated.

## Results and discussion

### Chemistry

A series of myricetin derivatives containing thiadiazole moiety were successfully prepared in two steps in our current work. All of the target compounds **2**, **3a**–**3q** were characterized by infrared spectrum (IR), nuclear magnetic resonance (NMR) spectroscopy, and high resolution mass spectrum (HRMS) analysis. The IR spectral data of compounds **2**, **3a**–**3q** showed characteristic frequencies at 1723–1709 cm^−1^ and 1640–1621 cm^−1^, which are assigned to the characteristic vibrations of C=O and C=N–, respectively. In the ^1^H NMR spectra, the characteristic −CH_2_—groups between myricetin scaffold and 1,3,4-thiadiazole heterocycle was observed as a signal at approximately 5.27–5.21 ppm. The chemical shifts at 165.59–161.63 and 161.70–154.04 ppm in the ^13^C NMR spectra confirmed the existence of C=O and C=N-groups, respectively.

### Antibacterial activity screening of the title compounds against Xac and *Rs* in vitro

Using *Ralstonia solanacearum* (strain MR111, Guizhou University, China) and *Xanthomonas oryzae pv. oryzae* (strain PXO99A, Nanjing Agricultural University, China) as the tested bacterial strains, the antibacterial activities of title compounds have been evaluated by the turbidimeter test [1, 3, 4, 6], and the commercial agent *thiadiazole*-*copper* was tested as the control. Some compounds with good antibacterial activity against *Xoo* and *Rs* were tested at five double-declining concentrations (100, 50, 25, 12.5 and 6.25 μg/mL) to obtain the corresponding EC_50_ values.

The title compounds (**2**, **3a**–**3q**) were evaluated for antibacterial activities against *Xoo* and *Rs* in vitro. Results in Table 1 indicated that most synthesized compounds exhibited appreciable antibacterial activities against *Xoo* and *Rs*. For example, compounds **2**, **3a**, **3b**, **3d**, **3f**, **3i**, **3m** and **3p** showed excellent antibacterial activities against *Xoo* at 100 μg/mL, with inhibition rates of 84.5, 84.9, 99.6, 87.3, 77.5, 84.5, 99.3 and 84.3%, respectively, which were better than that of *thiadiazole*-*copper* (52.3%). The inhibition rates of compounds **2**, **3a**, **3b**, **3d**, **3f**, **3i**, **3m** and **3p** against *Xoo* at 50 μg/mL were 54.6, 60.1, 65.2, 90.7, 82.6, 68.2, 80.8 and 71.2%, respectively, which were better than that of *thiadiazole*-*copper* (28.7%). Additionally, compounds **3b**, **3d**, **3e**, **3f**, **3i** and **3o** demonstrated good antibacterial activities against *Rs* at 100 μg/mL, with inhibition rates of 81.4, 64.3, 75.7, 69.3, 64.3 and 65.4%, respectively, which were superior to that of *thiadiazole*-*copper* (46.7%). Compounds **3b**, **3d**, **3e**, **3f**, **3i** and **3o** showed good antibacterial activities against *Rs* at 50 μg/mL (60.2, 30.4, 65.5, 40.5, 52.2 and 52.1%, respectively), which were better than *thiadiazole*-*copper* (32.2%).

**Table 1:** Inhibition effect of the compounds **4**, **5a**–**5q** against *Xoo* and *Rs*

Compd.	R	*Xoo*		*Rs*	
		100 μg/mL	50 μg/mL	100 μg/mL	50 μg/mL
**2**	–	84.5 ± 3.9	54.6 ± 8.5	46.5. ± 9.7	28.1 ± 7.8
**3a**	H	84.9 ± 5.8	60.1 ± 2.5	36.0 ± 2.6	32.4 ± 6.1
**3b**	4-NO_2_Ph	81.4 ± 4.6	65.2 ± 9.0	81.5 ± 6.7	60.2 ± 6.9
**3c**	2-MePh	47.2 ± 1.5	25.9 ± 3.7	49.3 ± 6.7	30.3 ± 3.8
**3d**	4-ClPh	99.6 ± 0.1	90.7 ± 4.0	64.3 ± 8.8	30.4 ± 4.1
**3e**	Me	58.2 ± 5.1	27.4 ± 5.4	75.7 ± 8.1	65.5 ± 9.9
**3f**	2-ClPh	87.3 ± 2.5	82.6 ± 2.6	69.3 ± 0.8	46.5 ± 9.1
**3g**	2-FPh	79.7 ± 3.6	21.0 ± 4.9	45.2 ± 5.9	38.3 ± 2.4
**3h**	4-OMePh	37.3 ± 6.2	15.5 ± 8.9	28.1 ± 7.6	27.1 ± 6.0
**3i**	2,4-di-ClPh	77.5 ± 1.4	68.2 ± 5.4	64.3 ± 6.1	52.1 ± 2.8
**3j**	3-NO_2_Ph	30.0 ± 1.2	79.8 ± 9.7	45.2 ± 8.3	31.1 ± 4.3
**3k**	4-BrPh	47.3 ± 4.7	23.3 ± 7.5	26.4 ± 2.6	10.7 ± 1.6
**3l**	2-BrPh	50.7 ± 1.9	31.6 ± 4.5	24.0 ± 4.7	16.2 ± 0.7
**3m**	2-Cl-thiazol-5-yl	99.4 ± 3.9	80.8 ± 3.7	26.3 ± 3.2	25.0 ± 6.6
**3n**	Ph	38.3 ± 4.5	17.7 ± 0.1	45.3 ± 5.6	44.7 ± 5.1
**3o**	4-MePh	52.6 ± 3.3	37.6 ± 5.5	65.4 ± 1.7	52.1 ± 5.7
**3p**	Pyridin-3-yl	84.3 ± 3.8	71.2 ± 5.3	38.0 ± 6.2	12.8 ± 6.0
*Myricetin* ^a^	–	40.1 ± 8.3	21.0 ± 5.6	28.6 ± 2.2	17.5 ± 3.3
*Thiadiazole*-*copper* ^a^	–	52.4 ± 2.0	28.7 ± 4.1	46.7 ± 2.0	32.2 ± 2.1

Average of three replicates

^a^ *Thiadiazole*-*copper* and *myricetin* were used for comparison of antibacterial activity

To further understand antibacterial activity of synthesized compounds, the EC_50_ values of some target compounds, which exhibited better antibacterial activities against *Xoo* and *Rs* than *thiadiazole*-*copper*, were calculated and summarized in Table 2. Notably, compounds **2**, **3a**, **3b**, **3d**, **3f**, **3i**, **3m** and **3p** exhibited excellent antibacterial activities against Xoo, with EC_50_ values of 42.7, 38.6, 20.8, 12.9, 22.7, 27.3, 18.3 and 29.4 μg/mL, respectively, which were better than that of *thiadiazole*-*copper* (94.9 μg/mL). Meanwhile, compounds **3b**, **3d**, **3e**, **3f**, **3i** and **3o** showed remarkable antibacterial activities against *Rs*, with EC_50_ values of 37.9, 72.6, 43.6, 59.6, 60.6 and 39.6 μg/mL, respectively, which were superior to that of *thiadiazole*-*copper* (131.7 μg/mL).

**Table 2:** EC_50_ values of target compounds against *Xoo* and *Rs*

Compd.	*Xoo*			*Rs*		
	Regression equation	r	EC_50_ (µg/mL)	Regression equation	r	EC_50_ (µg/mL)
**2**	y = 2.513x + 0.902	0.99	42.7 ± 2.6	/	/	/
**3a**	y = 2.885x + 0.454	0.99	38.6 ± 1.4	/	/	/
**3b**	y = 1.199x + 3.420	0.99	20.8 ± 3.6	y = 2.685x + 0.762	0.99	37.9 ± 1.0
**3d**	y = 2.328x + 2.418	0.97	12.9 ± 5.8	y = 2.770x-0.154	0.99	72.6 ± 1.6
**3e**	/	/	/	y = 2.485x + 0.925	0.98	43.6 ± 3.8
**3f**	y = 1.982x + 2.314	0.98	22.7 ± 3.6	y = 3.004x-0.332	0.99	59.6 ± 2.0
**3i**	y = 1.401x + 2.989	0.99	27.3 ± 1.8	y = 2.365x + 0.786	0.99	60.6 ± 2.1
**3m**	y = 2.723x + 1.565	0.98	18.3 ± 3.6	/	/	/
**3p**	y = 2.058x + 1.979	0.99	29.4 ± 1.0	/	/	/
**3o**	/	/	/	y = 1.017x + 3.375	0.96	39.6 ± 5.3
*Thiadiazole*-*copper* ^a^	y = 1.999x + 1.047	0.99	94.9 ± 2.2	y = 0.930x + 3.028	0.98	131.7 ± 2.9

Average of three replicates

^a^The commercial agricultural antibacterial agent *thiadiazole*-*copper* was used for comparison of antibacterial activity

The inhibitory rates in Tables 1 and 2 indicated that most synthesized compounds bearing the same substituted fragment were found to exhibit better antibacterial activity against *Xoo* than *Rs*. For example, the EC_50_ values of title compounds **3b**, **3d**, **3f** and **3i** against *Xoo* were respectively 20.8, 12.9, 22.7 and 27.3 μg/mL, which were better than that against *Rs* (37.9, 72.6, 59.6 and 60.6 μg/mL, respectively). The antibacterial results in Tables 1 and 2 also indicated that the different groups on R had significant effects on the antibacterial activity of the target compounds. Obviously, the presence of heterocycles can effectively enhance the antibacterial activity against *Xoo*. As examples of this phenomenon, the compounds **3m** and **3p**, which contain respectively 2-Cl-thiazol-5-yl and pyridin-3-yl groups, exhibited fine antibacterial activities against *Xoo* at 50 μg/mL, with the inhibition rates of 80.8 and 71.2%, respectively, which were superior to that of *thiadiazole*-*copper* (28.7%). Meanwhile, when R was substituted with 4-NO_2_Ph, 4-ClPh, 2-ClPh and 2,4-di-ClPh groups, the corresponding compounds **3b**, **3d**, **3f** and **3i** exhibit remarkable antibacterial activities against *Xoo*, with the EC_50_ values of 20.8, 12.9, 22.7 and 27.3 μg/mL, respectively, which were better than that of *thiadiazole*-*copper* (94.9 μg/mL).

### Antiviral activity screening of the title compounds against TMV in vivo

Using growing *N. tobacum L.* leaves at the same age as the test subjects, the curative and protective activities against TMV were evaluated based on the half-leaf blight spot method [25–27], and the commercial agent *ningnanmycin* was tested as the control under the same conditions. The antiviral activity against TMV in vivo at 500 μg/mL was listed in Tables 3 and 4. The preliminary bioassays results indicated that the inhibitory rates of title compounds against TMV at 500 μg/mL ranged from 18.2 to 68.4% in terms of their curative activity, and ranged from 21.5 to 60.8% in terms of their protective activity. Among them, the inhibitory rates of compounds **3d**, **3f**, **3i** and **3m** in curative activity were 59.8, 68.4, 66.8 and 57.1%, respectively, which were better than that of *ningnanmycin* (51.8%). Moreover, compounds **3c**, **3i** and **3m** were found to exhibit significant protective activities (58.4, 60.8 and 56.7%, respectively), which were similar to *ningnanmycin* (58.3%).

**Table 3:** Antiviral activities of the title compounds against TMV in vivo at 500 μg/mL

Compd.	Curative activity (%)	Protection activity (%)	Compd.	Curative activity (%)	Protection activity (%)
**2**	18.2 ± 7.3	21.5 ± 9.1	**3j**	28.7 ± 3.8	39.4 ± 3.1
**3a**	46.7 ± 5.2	50.3 ± 9.3	**3k**	28.0 ± 8.6	33.0 ± 7.5
**3b**	53.8 ± 9.0	54.1 ± 9.4	**3l**	33.9 ± 9.4	34.2 ± 5.4
**3c**	37.0 ± 9.1	58.4 ± 1.0	**3m**	57.1 ± 9.6	56.7 ± 8.2
**3d**	59.8 ± 9.2	54.3 ± 9.0	**3n**	48.4 ± 5.9	42.1 ± 7.1
**3e**	28.7 ± 8.3	35.4 ± 5.1	**3o**	50.8 ± 3.6	47.3 ± 2.9
**3f**	68.4 ± 7.4	54.4 ± 7.7	**3p**	34.6 ± 5.4	36.5 ± 1.6
**3g**	36.4 ± 3.8	38.6 ± 7.7	*Myricetin* ^a^	28.8 ± 6.7	34.4 ± 7.2
**3** **h**	44.8 ± 9.4	45.2 ± 1.5	*Ningnanmycin* ^a^	51.8 ± 4.3	58.3 ± 2.9
**3i**	66.8 ± 9.8	60.8 ± 8.3			

Average of three replicates

^a^N*ingnanmycin* and *myricetin* were used for comparison of antiviral activity

**Table 4:** The EC_50_ values of **5d**, **5f**, **5i** and **5m** against TMV

Compd.	TMV		Regression equation	r	EC_50_ (µg/mL)
	500 μg/mL	250 μg/mL			
**3d**	59.8 ± 6.2	55.2 ± 4.4	y = 0.473x − 3.967	0.98	152.8 ± 3.2
**3f**	68.4 ± 7.4	64.2 ± 8.8	y = 0.744x − 3.512	0.99	99.7 ± 2.7
**3i**	66.8 ± 9.8	63.3 ± 5.8	y = 0.816x + 3.823	0.99	127.1 ± 2.6
**3m**	57.1 ± 9.6	52.3 ± 8.5	y = 0.361x + 4.197	0.99	167.3 ± 4.8
*Ningnanmycin* ^a^	51.3 + 2.6	50.3 + 3.8	y = 0.203x + 4.154	0.97	211.1 ± 3.6

Average of three replicates

^a^The commercial agricultural antiviral agent *ningnanmycin* was used for comparison of antiviral activity

To further understand antiviral activity of synthesized compounds, the EC_50_ values of **3d**, **3f**, **3i** and **3m** were calculated and summarized in Table 4. Notably, the EC_50_ values of **3d**, **3f**, **3i** and **3m** were respectively 152.8, 99.7, 127.1 and 167.3 μg/mL, which were better than that of *ningnanmycin* (211.1 μg/mL).

The antiviral results in Tables 3 and 4 indicated that most of synthesized compounds bearing the same substituted fragment exhibited better protective activity than curative activity against TMV. Meanwhile, Results in Tables 3 and 4 also indicated that the different groups on R had significant effects on the anti-TMV activity of the target compounds. Obviously, the presence of benzyl chloride groups can effectively enhance the curative activity of title compounds against TMV. For example, compounds **3d**, **3f**, **3i** and **3m,** which contain respectively 2-ClPh, 4-ClPh, 2,4-di-ClPh and 2-Cl-thiazol-5-yl groups, exhibited excellent curative activities against TMV, with the EC_50_ values of 152.8, 99.7, 127.1 and 167.3 μg/mL, respectively, which were better than that of *ningnanmycin* (211.1 μg/mL). Furthermore, when the R was 2-MePh, 2,4-di-ClPh and 2-Cl-thiazol-5-yl groups, the protective activities of corresponding compounds **3c**, **3i** and **3m** at 500 μg/mL were 58.4, 60.8 and 56.7%, respectively, which were similar to that of *ningnanmycin* (58.3%).

## Methods and materials

### Chemistry

The melting points of the products were determined on an XT-4 binocular microscope (Beijing Tech Instrument Co.). The ^1^H NMR and ^13^C NMR (CDCl_3_ or DMSO as solvents) spectroscopies were performed on a JEOL-ECX 500 NMR spectrometer at room temperature using TMS as an internal standard. The IR spectra were recorded on a Bruker VECTOR 22 spectrometer using KBr disks. High-performance liquid chromatography mass spectrometry was performed on a Thermo Scientific Q Exactive (USA). Unless noted, all solvents and reagents were purchased from Shanghai Titan Scientific Co., Ltd, and were treated with standard methods. Based on the synthesis procedures described in our previous work [14], intermediates **1** (2-((5,7-dimethoxy-4-oxo-2-(3,4,5-trimethoxyphenyl)-4H-chromen-3-yl)oxy)aceto-hydrazide) were prepared using myricetrin (5,7-dihydroxy-3-(3,4,5-trihydroxy-6-methyltetrahydro-2H-pyran-2-yl)oxy)-2-(3,4,5-trihydroxyphenyl)-4H-chromen-4-one) as the starting material.

### General synthesis procedure for 5,7-dimethoxy-2-(3,4,5-trimethoxyphenyl)-3- ((5-mercapto-1,3,4-thiadiazol-2-yl)methoxy)-4H-chromen-4-one (**2**)

To a solution of intermediate **1** (1.00 g, 2.17 mmol) in methanol (30 mL), potassium hydroxide (0.20 mL, 3.16 mmol) and carbon disulfide (0.21 mL, 3.47 mmol) were added, and the reaction mixture was heated under reflux for 16 h. After the reaction was cooled to room temperature, 50 mL of water was added to the mixture, and the pH of the solution was adjusted to five with dilute HCl. Then, a solid precipitated was filtered and recrystallized with ethanol to obtain the intermediate **2**. white solid, m. p. 154–155 °C, yield 50.1%; IR (KBr, cm^−1^): 3229, 2939, 2837, 1639, 1634, 1608, 1575, 1498, 1466, 1357, 1253, 1211, 1130, 944, 816; ^1^H NMR (500 MHz, DMSO-*d*_*6*_) δ 7.24 (s, 2H, Ar–H), 6.87 (d, *J* = 2.1 Hz, 1H, Ar–H), 6.53 (d, *J* = 2.1 Hz, 1H, Ar–H), 5.09 (s, 2H, CH_2_), 3.91 (s, 3H, OCH_3_), 3.86 (s, 9H, 3 OCH_3_), 3.77 (s, 3H, OCH_3_); ^13^C NMR (125 MHz, DMSO-*d*_*6*_) δ 183.1, 176.9, 169.4, 165.6, 164.6, 163.5, 158.2, 157.9, 145.03, 143.4, 129.9, 113.5, 111.2, 101.5, 98.6, 67.3, 65.5, 61.5, 61.4, 61.3; HRMS (HPLC) *m/z*: 519.0890, found 519.0883 ([M+H]^+^).

### General synthesis procedures for title compounds **3a**–**3p**

To a solution of **2** (1.16 mmol) in acetonitrile (30 mL), sodium carbonate (1.74 mmol) and CH_3_I (1.74 mmol) were added, and the reaction mixture was stirred at 40 °C for 5 h. After the reaction was completed and cooled to room temperature, a solid precipitated was filtered and recrystallized with methanol to obtain the title compound **3a**. Based on the similar method, the title compounds **3b**–**3p** were prepared.

### 5,7-Dimethoxy-2-(3,4,5-trimethoxyphenyl)-3-((5-(methylthio)-1,3,4-thiadiazol-2-yl)methoxy)-4H-chromen-4-one **(3a)**

A white solid, m. p. 183–184 °C, yield 50.3%; IR (KBr, cm^−1^): 3006, 2957, 2839, 1645, 1616, 1580, 1474, 1427, 1417, 1212, 1163, 1158, 993, 819, 768; ^1^H NMR (500 MHz, CDCl_3_) δ 7.10 (s, 2H, Ar–H), 6.47 (s, 1H, Ar–H), 6.34 (s, 1H, Ar–H), 5.23 (s, 2H, CH_2_), 3.95 (s, 3H, OCH_3_), 3.90-3.87 (m, 12H, 4 OCH_3_), 2.56 (s, 3H, CH_3_); ^13^C NMR (125 MHz, CDCl_3_) δ 173.3, 166.6, 164.4, 163.3, 161.1, 159.0, 154.3, 152.9, 140.1, 138.6, 125.1, 109.3, 106.1, 96.2, 92.7, 62.3, 61.03, 56.5, 56.4, 56.9, 14.4; HRMS (HPLC) *m/z*: 555.0866, found 555.0837 ([M+Na]^+^).

### 5,7-Dimethoxy-2-(3,4,5-trimethoxyphenyl)-3-((5-((4-nitrobenzyl)thio)-1,3,4-thiadiazol-2-yl)methoxy)-4H-chromen-4-one **(3b)**

A yellow solid, m. p. 124–125 °C, yield 30.1%; IR (KBr, cm^−1^): 2942, 1700, 1637,1604, 1575, 1519, 1471, 1455, 1349, 1362, 1243, 1211, 1164, 1126, 1108, 1017, 856, 821; ^1^H NMR (500 MHz, DMSO-*d*_6_) δ 8.13 (d, *J* = 8.7 Hz, 2H, Ar–H), 7.62 (d, *J* = 8.7 Hz, 2H, Ar–H), 7.18 (s, 2H, Ar–H), 6.82 (d, *J* = 2.1 Hz, 1H, Ar–H), 6.50 (d, *J* = 2.1 Hz, 1H, Ar–H), 5.21 (s, 2H, CH_2_), 4.48 (s, 2H, CH_2_), 3.87 (s, 3H, OCH_3_), 3.83 (s, 3H, OCH_3_), 3.77 (s, 6H, 2 OCH_3_), 3.70 (s, 3H, OCH_3_); ^13^C NMR (125 MHz, DMSO-*d*_*6*_) δ 172.1, 164.6, 164.5, 164.2, 160.9, 158.8, 153.2, 153.1, 147.4, 145.1, 140.2, 138.6, 130.8, 128.5, 125.2, 124.6, 124.1, 108.8, 106.4, 96.8, 93.8, 62.3, 60.7, 56.7, 56.6, 56.5, 35.2; HRMS (HPLC) m/z: 676.1030, found 676.0.0985 ([M+Na]^+^).

### 5,7-Dimethoxy-2-(3,4,5-trimethoxyphenyl)-3-((5-((2-methylbenzyl)thio)-1,3,4-thiadiazol-2-yl)methoxy)-4H-chromen-4-one **(3c)**

A white solid, m. p. 155–157 °C, yield 54.3%; IR (KBr, cm^−1^): 3010, 2954, 2838, 1649, 1610, 1572, 1511, 1470, 1452, 1424, 1356, 1211, 1194, 1181,1166, 1126, 1058, 1019, 978,949, 827, 817; ^1^H NMR (500 MHz, CDCl_3_) δ 7.26 (s, 1H, Ar–H), 7.25 (s, 1H, Ar–H), 7.14 (s, 2H, Ar–H), 7.11 (d, *J* = 7.8 Hz, 2H, Ar–H), 6.49 (d, *J* = 2.2 Hz, 1H, Ar–H), 6.37 (d, *J* = 2.2 Hz, 1H, Ar–H), 5.27 (s, 2H, CH_2_), 4.31 (s, 2H, CH_2_), 3.97 (s, 3H, OCH_3_), 3.91 (s, 3H, OCH_3_), 3.90 (s, 3H, OCH_3_), 3.88 (s, 6H, 2 OCH_3_), 2.31 (s, 3H, CH_3_); ^13^C NMR (125 MHz, CDCl_3_) δ 173.3, 165.7, 164.4, 163.3, 161.2, 159.0, 154.1, 153.0, 140.3, 138.7, 138.1, 132.0, 129.6, 129.2, 125.1, 109.4, 106.1, 96.2, 92.7, 62.4, 61.1, 56.6, 56.4, 56.0, 36.5, 29.8, 21.3; HRMS (HPLC) m/z: 645.1335, found 645.1330 ([M+Na]^+^).

### 5,7-Dimethoxy-2-(3,4,5-trimethoxyphenyl)-3-((5-((4-chlorobenzyl)thio)-1,3,4-thiadiazol-2-yl)methoxy)-4H-chromen-4-one **(3d)**

A white solid; m. p. 127–128 °C; yield, 60.1%; IR (KBr, cm^−1^): 3003, 2947, 2838, 1652, 1633, 1613, 1578, 1492, 1477, 1469, 1416, 1356, 1241, 1212, 1132, 1058, 1017, 948, 839, 814; ^1^H NMR (500 MHz, CDCl_3_) δ 7.33 (t, *J* = 5.7 Hz, 2H, Ar–H), 7.27 (d, *J* = 1.6 Hz, 1H, Ar–H), 7.14 (s, 2H, Ar–H), 6.50 (d, *J* = 2.0 Hz, 1H, Ar–H), 6.38 (d, *J* = 2.0 Hz, 1H, Ar–H), 5.27 (s, 2H, CH_2_), 4.30 (s, 2H, CH_2_), 3.98 (s, 3H, OCH_3_), 3.91 (d, *J* = 2.7 Hz, 6H, 2 OCH_3_), 3.89 (s, 6H, 2 OCH_3_); ^13^C NMR (125 MHz, CDCl_3_) δ 173.3, 165.2, 164.4, 163.5, 161.2, 159.0, 154.1, 152.9, 140.2, 138.7, 134.1, 133.9, 130.6, 129.0, 125.1, 109.4, 106.1, 96.2, 92.7, 62.4, 61.1, 56.6, 56.4, 56.0, 35.9; HRMS (HPLC) *m/z*: 665.0789, found 665.0746 ([M+Na]^+^).

### 5,7-Dimethoxy-2-(3,4,5-trimethoxyphenyl)-3-((5-(ethylthio)-1,3,4-thiadiazol-2-yl)methoxy)-4H- chromen-4-one **(3e)**

A white solid, m. p. 187–188 °C; yield 35.3%; IR (KBr, cm^−1^): 2953, 2836, 1645, 1634, 1580, 1492, 1472, 1452, 1414, 1357, 1213, 1169, 1123, 1105, 992, 817; ^1^H NMR (500 MHz, DMSO-*d*_*6*_) δ 7.18 (s, 2H, Ar–H), 6.81 (s, 1H, Ar–H), 6.49 (s, 1H, Ar–H), 5.22 (s, 2H, CH_2_), 3.87 (s, 3H, OCH_3_), 3.83 (s, 3H, OCH_3_), 3.80 (s, 6H, 2 OCH_3_), 3.72 (s, 3H, OCH_3_), 3.07 (q, *J* = 6.8 Hz, 2H, CH_2_), 1.24 (t, *J* = 4.5 Hz, 3H, CH_3_); ^13^C NMR (125 MHz, DMSO-*d*_6_) δ 172.1, 165.3, 164.6, 163.7, 160.9, 158.8, 153.3, 153.1, 140.2, 138.5, 125.2, 108.8, 106.3, 96.7, 93.8, 62.2, 60.7, 56.7, 56.6, 56.5, 26.9, 15.1; HRMS (HPLC) *m/z*: 569.1022, found 569.0983 ([M+Na]^+^).

### 5,7-Dimethoxy-2-(3,4,5-trimethoxyphenyl)-3-((5-((2-chlorobenzyl)thio)-1,3,4-thiadiazol-2-yl)methoxy)-4H-chromen-4-one **(3f)**

A white solid, m. p. 112–113 °C; yield 36.6%; IR (KBr, cm^−1^): 2997, 2942, 2838, 1636, 1603, 1578, 1572, 1505, 1490, 1470, 1454, 1415, 1350, 1245, 1211, 1164, 1127, 1108, 1018, 1003, 853, 820; ^1^H NMR (500 MHz, CDCl_3_) δ 7.52 (d, *J* = 7.4 Hz, 1H, Ar–H), 7.38–7.34 (m, 1H, Ar–H), 7.20 (m, 2H, Ar–H), 7.15 (s, 2H, Ar–H), 6.49 (d, *J* = 2.2 Hz, 1H, Ar–H), 6.37 (d, *J* = 2.1 Hz, 1H, Ar–H), 5.28 (s, 2H, CH_2_), 4.45 (s, 2H, CH_2_), 3.97 (s, 3H, OCH_3_), 3.91 (s, 3H, OCH_3_), 3.90 (s, 3H, OCH_3_), 3.87 (s, 6H, 2 OCH_3_); ^13^C NMR (125 MHz, CDCl_3_) δ 173.3, 165.5, 164.4, 163.6, 161.2, 159.0, 154.0, 153.0, 140.2, 138.7, 134.4, 133.5, 131.6, 129.8, 129.7, 127.2, 125.1, 109.4, 106.0, 96.2, 92.6, 62.4, 61.1, 56.8, 56.4, 56.0, 34.5; HRMS (HPLC) *m/z*: 665.0789, found 665.0747 (([M+Na]^+^).

### 5,7-Dimethoxy-2-(3,4,5-trimethoxyphenyl)-3-((5-((2-fluorobenzyl)thio)-1,3,4-thiadiazol-2-yl)methoxy)-4H-chromen-4-one **(3g)**

A white solid, m. p. 124–125 °C, yield 70.4%; IR (KBr, cm^−1^): 2975, 2942, 2842, 1637, 1604, 1492, 1470, 1455, 1415, 1350, 1244, 1212, 1167, 1167, 1126, 1106, 1017, 1005, 855; ^1^H NMR (500 MHz, CDCl_3_) δ 7.44 (t, *J* = 7.6 Hz, 1H, Ar–H), 7.25 (d, *J* = 1.3 Hz, 1H, Ar–H), 7.14 (s, 2H, Ar–H), 7.09–6.98 (m, 2H, Ar–H), 6.48 (s, 1H, Ar–H), 6.36 (s, 1H, Ar–H), 5.28 (s, 2H, CH_2_), 4.37 (s, 2H, CH_2_), 3.96 (s, 3H, OCH_3_), 3.90 (s, 6H, 2 OCH_3_), 3.87 (s, 6H, 2 OCH_3_); ^13^C NMR (125 MHz, CDCl_3_) δ 173.3, 165.4, 164.4, 163.5, 161.2, 160.3, 159.8, 159.0, 154.1, 153.0, 140.3, 138.7, 131.5, 130.2, 125.1, 124.4, 122.8, 115.8, 115.6, 109.4, 106.1, 96.2, 92.7, 62.4, 61.0, 56.5, 56.0, 29.9; HRMS (HPLC) *m/z*: 649.1085, found 649.1046 ([M+Na]^+^).

### 5,7-Dimethoxy-2-(3,4,5-trimethoxyphenyl)-3-((5-((4-methoxybenzyl)thio)-1,3,4-thiadiazol-2-yl)methoxy)-4H-chromen-4-one **(3h)**

A white solid, m. p. 146–147 °C, yield 35.7%; IR (KBr, cm^−1^): 2950, 1755, 1645, 1629, 1604, 1507, 1492, 1457, 1430, 1410, 1354, 1249, 1210, 1180, 1161, 1129, 1112, 1064, 1016, 841, 816; ^1^H NMR (500 MHz, CDCl_3_) δ 7.27 (d, *J* = 8.1 Hz, 2H, Ar–H), 7.19 (s, 1H, Ar–H), 6.83 (d, *J* = 7.5 Hz, 4H, Ar–H), 6.50 (s, 1H, Ar–H), 5.23 (s, 2H, CH_2_), 4.29 (s, 2H, CH_2_), 3.87 (s, 3H, OCH_3_), 3.83 (s, 3H, OCH_3_), 3.78 (s, 6H, 2 OCH_3_), 3.70 (s, 3H, OCH_3_), 3.69 (s, 3H, OCH_3_); ^13^C NMR (125 MHz, CDCl_3_) δ 172.2, 167.0, 164.6, 163.9, 160.9, 159.4, 158.8, 153.1, 140.2, 138.6, 130.9, 128.4, 125.3, 114.5, 114.0, 108.8, 106.4, 96.7, 93.8, 63.1, 62.3, 60.7, 56.6, 55.6, 35.9; HRMS (HPLC) *m/z*: 639.1447, found 639.1444 ([M+H]^+^).

### 5,7-Dimethoxy-2-(3,4,5-trimethoxyphenyl)-3-((5-((2,4-dichlorobenzyl)thio)-1,3,4-thiadiazol-2-yl)methoxy)-4H-chromen-4-one **(3i)**

A white solid, m. p. 154–155 °C, yield 90.1%; IR (KBr, cm^−1^): 2944, 1643, 1616, 1571, 1460, 1416, 1355, 1242, 1216, 1162, 1135, 1058, 1018, 955, 827; ^1^H NMR (500 MHz, CDCl_3_) δ 7.51 (d, *J* = 8.3 Hz, 1H, Ar–H), 7.38 (d, *J* = 2.1 Hz, 1H, Ar–H), 7.17 (d, *J* = 8.3 Hz, 1H, Ar–H), 7.14 (s, 2H, Ar–H), 6.50 (d, *J* = 2.1 Hz, 1H, Ar–H), 6.38 (d, *J* = 2.1 Hz, 1H, Ar–H), 5.28 (s, 2H, CH_2_), 4.40 (s, 2H, CH_2_), 3.98 (s, 3H, OCH_3_), 3.91 (s, 6H, 2 OCH_3_), 3.88 (s, 6H, 2 OCH_3_); ^13^C NMR (125 MHz, CDCl_3_) δ 173.3, 165.2, 164.4, 163.7, 161.2, 159.0, 154.0, 153.0, 138.7, 135.1, 134.9, 132.4, 132.2, 129.6, 127.5, 125.1, 109.4, 106.1, 96.2, 92.7, 62.4, 61.1, 56.6, 56.4, 56.0, 33.8; HRMS (HPLC) m/z: 699.0399, found 699.0365 ([M+Na]^+^).

### 5,7-Dimethoxy-2-(3,4,5-trimethoxyphenyl)-3-((5-((3-nitrobenzyl)thio)-1,3,4-thiadiazol-2-yl)methoxy)-4H-chromen-4-one **(3j)**

A white solid, m. p. 180–181 °C, yield 50.5%; IR(KBr, cm^−1^): 2942, 1700, 1637, 1604, 1575, 1519, 1471, 1455, 1349, 1362, 1243, 1211, 1164, 1126, 1108, 1017, 856, 821; ^1^H NMR (500 MHz, CDCl_3_) δ 8.10 (d, *J* = 8.1 Hz, 1H, Ar–H), 7.75 (d, *J* = 7.6 Hz, 1H, Ar–H), 7.56 (t, *J* = 7.5 Hz, 1H, Ar–H), 7.49–7.43 (m, 1H, Ar–H), 7.14 (s, 2H, Ar–H), 6.50 (d, *J* = 2.1 Hz, 1H, Ar–H), 6.37 (d, *J* = 2.2 Hz, 1H,Ar–H), 5.27 (s, 2H, CH_2_), 4.68 (s, 2H, CH_2_), 3.97 (s, 3H, OCH_3_), 3.91 (s, 3H, OCH_3_), 3.89 (s, 3H, OCH_3_), 3.86 (s, 6H, 2 OCH_3_); ^13^C NMR (125 MHz, CDCl_3_) δ 173.3, 165.7, 164.4, 163.8, 161.2, 159.0, 154.0, 153.0, 147.6, 140.3, 138.8, 134.1, 133.1, 132.5, 129.4, 125.7, 125.1, 109.4, 106.1, 96.2, 92.7, 62.4, 61.0, 56.6, 56.4, 56.0, 34.2; HRMS (HPLC) *m/z*: 676.1030, found 676.1012 ([M+Na]^+^).

### 5,7-Dimethoxy-2-(3,4,5-trimethoxyphenyl)-3-((5-((4-bromobenzyl)thio)-1,3,4-thiadiazol-2-yl)methoxy)-4H-chromen-4-one **(3k)**

A white solid, m. p. 131–132 °C; yield, 39.4%; IR (KBr, cm^−1^): 2945, 1634, 1605, 1558, 1471, 1426, 1352, 1246, 1212, 1163, 1130, 1018, 820; ^1^H NMR (500 MHz, CDCl_3_) δ 7.43 (d, *J* = 8.3 Hz, 2H, Ar–H), 7.28 (s, 1H, Ar–H), 7.25 (s, 1H, Ar–H), 7.13 (s, 2H, Ar–H), 6.49 (d, *J* = 2.2 Hz, 1H, Ar–H), 6.38 (d, *J* = 2.2 Hz, 1H, Ar–H), 5.26 (s, 2H, CH_2_), 4.27 (s, 2H, CH_2_), 3.98 (s, 3H, OCH_3_), 3.91 (s, 6H, 2 OCH_3_), 3.88 (s, 6H, 2 OCH_3_); ^13^C NMR (125 MHz, CDCl_3_) δ 173.3, 165.2, 164.4, 163.5, 161.2, 159.0, 154.1, 152.9, 140.2, 138.7, 134.4, 132.0, 131.0, 125.1, 122.3, 109.4, 106.1, 96.2, 92.7, 62.4, 61.1, 56.6, 56.4, 56.0, 35.9; HRMS (HPLC) *m/z*: 709.0293, found 709.0237 ([M+Na]^+^).

### 5,7-Dimethoxy-2-(3,4,5-trimethoxyphenyl)-3-((5-((2-bromobenzyl)thio)-1,3,4-thiadiazol-2-yl)methoxy)-4H-chromen-4-one **(3l)**

A white solid, m. p. 116–117 °C, yield 45.4%; IR (KBr, cm^−1^): 3004, 2943, 1633, 1603, 1560, 1545, 1492, 1467, 1428, 1416, 1353, 1247, 1213, 1166, 1112, 1126, 1109, 1018, 1005, 862, 815; ^1^H NMR (500 MHz, CDCl_3_) δ 7.57–7.52 (m, 2H, Ar–H), 7.23 (t, *J* = 7.5 Hz, 1H, Ar–H), 7.16–7.11 (m, 3H, Ar–H), 6.49 (d, *J* = 2.2 Hz, 1H, Ar–H), 6.37 (d, *J* = 2.2 Hz, 1H, Ar–H), 5.28 (s, 2H, CH_2_), 4.46 (s, 2H, CH_2_), 3.97 (s, 3H, OCH_3_), 3.90 (d, *J* = 1.0 Hz, 6H, 2 OCH_3_), 3.87 (s, 6H, 2 OCH_3_); ^13^C NMR (125 MHz, CDCl_3_) δ 172.2, 164.6, 164.4, 164.2, 160.9, 158.8, 153.3, 153.1, 140.1, 138.6, 135.5, 133.4, 132.0, 130.7, 128.6, 125.3, 124.5, 108.8, 106.4, 96.7, 93.8, 62.3, 60.7, 56.7, 56.6, 56.5, 37.1; HRMS (HPLC) *m/z*: 709.0284, found 709.0246 ([M+Na]^+^).

### 5,7-Dimethoxy-2-(3,4,5-trimethoxyphenyl)-3-((5-(((2-chlorothiazol-5-yl)methyl)thio)-1,3,4-thiadiazol-2-yl)methoxy)-4H-chromen-4-one **(3m)**

A white solid, m. p. 120–121 °C, yield 58.3%; IR (KBr, cm^−1^): 2996, 2945, 1645, 1634, 1606, 1572, 1506, 1484, 1456, 1414, 1352, 1242, 1212, 1164, 1130, 1106, 1050, 870, 821; ^1^H NMR (500 MHz, DMSO-*d*_*6*_) δ 7.56 (s, 1H, Ar–H), 7.19 (s, 2H, Ar–H), 6.83 (s, 1H, Ar–H), 6.50 (s, 1H, Ar–H), 5.24 (s, 2H, CH_2_), 4.61 (s, 2H, CH_2_), 3.87 (s, 3H, OCH_3_), 3.83 (s, 3H, OCH_3_), 3.78 (s, 6H, 2 OCH_3_), 3.69 (s, 3H, OCH_3_); ^13^C NMR (125 MHz, DMSO-*d*_*6*_) δ 172.1, 164.6, 164.5, 164.4, 160.9, 158.8, 153.3, 153.1, 151.1, 141.8, 140.1, 138.6, 137.8, 125.2, 108.8, 106.4, 96.8, 93.8, 62.3, 60.7, 56.7, 56.6, 56.5, 28.4; HRMS (HPLC) *m/z*: 672.0306, found 672.0262 ([M+Na]^+^).

### 5,7-Dimethoxy-2-(3,4,5-trimethoxyphenyl)-3-((5-(benzylthio)-1,3,4-thiadiazol-2-yl)methoxy)-4H- chromen-4-one **(3n)**

A white solid, m. p. 160–161 °C, yield 35.7%; IR (KBr, cm^−1^): 2979, 2942, 1634, 1602, 1579, 1505, 1492, 1470, 1454, 1416, 1351, 1246, 1211, 1163, 1128, 1108, 1000, 823; ^1^H NMR (500 MHz, DMSO-*d*_*6*_) δ 7.34 (d, *J* = 6.9 Hz, 2H, Ar–H), 7.25 (d, *J* = 10.3 Hz, 3H, Ar–H), 7.18 (s, 2H, Ar–H), 6.82 (t, *J* = 4.6 Hz, 1H, Ar–H), 6.49 (d, *J* = 2.1 Hz, 1H, Ar–H), 5.22 (s, 2H, CH_2_), 4.34 (s, 2H, CH_2_), 3.87 (s, 3H, OCH_3_), 3.83 (s, 3H, OCH_3_), 3.79 (d, *J* = 13.8 Hz, 6H, 2 OCH_3_), 3.70 (d, *J* = 7.8 Hz, 3H, OCH_3_); ^13^C NMR (125 MHz, CDCl_3_) δ 172.2, 164.9, 164.6, 164.0, 160.9, 158.8, 153.3, 153.1, 140.1, 138.6, 136.6, 129.5, 129.1, 128.4, 125.3, 108.8, 106.4, 96.7, 93.8, 62.3, 60.7, 56.7, 56.6, 56.5, 36.1; HRMS (HPLC) *m/z*: 631.1179, found 631.1143 ([M+Na]^+^).

### 5,7-Dimethoxy-2-(3,4,5-trimethoxyphenyl)-3-((5-((4-methylbenzyl)thio)-1,3,4-thiadiazol-2-yl)methoxy)-4H-chromen-4-one **(3o)**

A white solid, m. p. 166–167 °C, yield 28.7%; IR (KBr, cm^−1^): 2933, 2838, 1649, 1610, 1578, 1511, 1470, 1410, 1357, 1239, 1121, 1160, 1126, 1019, 938, 817; ^1^H NMR (500 MHz, DMSO-*d*_*6*_) δ 7.23 (s, 1H, Ar–H), 7.21 (s, 1H, Ar–H), 7.18 (s, 2H, Ar–H), 7.07 (d, J = 7.9 Hz, 2H, Ar–H), 6.80 (d, J = 2.2 Hz, 1H, Ar–H), 6.48 (d, J = 2.2 Hz, 1H, Ar–H), 5.23 (s, 2H, CH_2_), 4.29 (s, 2H, CH_2_), 3.86 (s, 3H, OCH_3_), 3.82 (s, 3H, OCH_3_), 3.78 (s, 6H, 2 OCH_3_), 3.70 (s, 3H, OCH_3_), 2.22 (s, 3H, CH_3_); ^13^C NMR (125 MHz, DMSO-*d*_*6*_) δ 172.1, 164.9, 164.5, 163.9, 160.9, 158.7, 153.3, 153.1, 140.2, 138.6, 137.7, 133.4, 129.6, 129.4, 125.3, 108.8, 106.4, 96.7, 93.8, 62.3, 60.7, 56.7, 56.6, 56.5, 36.0, 21.2; HRMS (HPLC) *m/z*: 645.1335, found 645.1300 ([M+Na]^+^).

### 5,7-Dimethoxy-2-(3,4,5-trimethoxyphenyl)-3-((5-((pyridin-3-ylmethyl)thio)-1,3,4-thiadiazol-2-yl)methoxy)-4H-chromen-4-one **(3p)**

A white solid, m. p. 155–156 °C, yield 60.1%; IR (KBr, cm^−1^): 2943, 2839, 1633, 1622, 1602, 1505, 1470, 1464, 1428, 1351, 1247, 1212, 1166, 1128, 1109, 856, 817; ^1^H NMR (500 MHz, DMSO-*d*_*6*_) δ 8.56 (s, 1H, Ar–H), 8.43 (d, *J* = 4.5 Hz, 1H, Ar–H), 7.77 (d, *J* = 7.5 Hz, 1H, Ar–H), 7.35–7.24 (m, 1H, Ar–H), 7.18 (s, 2H, Ar–H), 6.82 (s, 1H, Ar–H), 6.50 (s, 1H, Ar–H), 5.21 (s, 2H, CH_2_), 4.38 (s, 2H, CH_2_), 3.87 (s, 3H, OCH_3_), 3.83 (s, 3H, OCH_3_), 3.77 (s, 6H, 2 OCH_3_), 3.70 (s, 3H, OCH_3_); ^13^C NMR (125 MHz, DMSO-*d*_*6*_) δ 172.1, 164.6, 164.6, 164.1, 160.9, 158.8, 153.3, 153.1, 150.5, 149.4, 140.1, 138.6, 137.1, 133.0, 125.3, 124.1, 108.8, 106.4, 96.7, 93.8, 62.3, 60.7, 56.7, 56.6, 56.5, 33.3; HRMS (HPLC) *m/z*: 632.1131, found 632.1095 ([M+Na]^+^).

## Conclusions

Aiming to discover novel myricetin analogues with potent activities, a series of novel *myricetin* derivatives containing 1,3,4-thiadiazole moiety were synthesized, and their antibacterial activities against *Xoo* and *Rs* and their antiviral activity against TMV were evaluated. Bioassays indicated that some target compounds exhibited potential antibacterial and antiviral activities. Among them, compounds **2**, **3a**, **3b**, **3d**, **3f**, **3i, 3m** and **3p** exhibited excellent antibacterial activities against *Xoo*, with EC_50_ values of 42.7, 38.6, 20.8, 12.9, 22.7, 27.3, 18.3 and 29.4 μg/mL, respectively, which were better than that of *thiadiazole*-*copper* (94.9 μg/mL). Compounds **3b**, **3d**, **3e**, **3f**, **3i** and **3o** showed good antibacterial activities against *Rs*, with EC_50_ values of 37.9, 72.6, 43.6, 59.6, 60.6 and 39.6 μg/mL, respectively, which were superior to that of *thiadiazole*-*copper* (131.7 μg/mL). In addition, compounds **3d**, **3f**, **3i** and **3m** showed better curative activities against TMV, with EC_50_ values of 152.8, 99.7, 127.1, and 167.3 μg/mL, respectively, which were better than that of *ningnanmycin* (211.1 μg/mL). Given the above results, this kind of myricetin analogues could be further studied as potential alternative templates in the search for novel antibacterial and antiviral agents.

## Additional file

**Additional file 1.** All the copies of IR, ^1^H NMR, ^13^C NMR and HRMS for the title compounds.
