# Supplementary material for: Synthesis and biological activity of myricetin derivatives containing 1,3,4-thiadiazole scaffold
Source: Chem Cent J. 2017 Oct 17;11:106. doi: 10.1186/s13065-017-0336-7 (PMC5645266; doi:10.1186/s13065-017-0336-7)
Supplement: Supplementary file 1 — Additional file 1. All the copies of IR, 1H NMR, 13C NMR and HRMS for the title compounds. [file 13065_2017_336_MOESM1_ESM.doc]

**Additional Information**

**Synthesis and biological activity of myricetin derivatives containing 1,3,4-thiadiazole scaffold**

Xinmin Zhong^1, ‡^, Xiaobin Wang^1, 2, ‡^, Lijuan Chen^1^, Xianghui Ruan^1^, Qin Li^1^, Juping Zhang^1^, Zhuo Chen^1^, Wei Xue^1^, *

**^1^** State Key Laboratory Breeding Base of Green Pesticide and Agricultural Bioengineering, Key Laboratory of Green Pesticide and Agricultural Bioengineering, Ministry of Education, Guizhou University, Guiyang 550025, China

**^2^** Key Laboratory of Monitoring and Management of Crop Diseases and Pest Insects, Ministry of Agriculture, Nanjing Agricultural University, Nanjing 210095, China

*Corresponding author: [wxue@gzu.edu.cn](../wxue@gzu.edu.cn) (Wei Xue)

^‡^: Xinmin Zhong and Xiaobin Wang contributed equally to this work.

**Table of** Contents

[Figure **S1**. IR spectrum of compound **2** 4](#__RefHeading___Toc10)

[Figure **S2**. ^1^H NMR spectrum of compound **2** 4](#__RefHeading___Toc12783)

[Figure **S3**. ^13^C NMR spectrum of compound **2** 4](#__RefHeading___Toc27620)

[Figure **S4**. HRMS spectrum of compound **2** 5](#__RefHeading___Toc5638)

[Figure **S5**. IR spectrum of compound **3a** 5](#__RefHeading___Toc31437)

[Figure **S6**. ^1^H NMR spectrum of compound **3a** 6](#__RefHeading___Toc8545)

[Figure **S7**. ^13^C NMR spectrum of compound **3a** 6](#__RefHeading___Toc29595)

[Figure **S8**. HRMS spectrum of compound **3a** 7](#__RefHeading___Toc7424)

[Figure **S9**. IR spectrum of compound **3b** 7](#__RefHeading___Toc10946)

[Figure **S10**. ^1^H NMR spectrum of compound **3b** 8](#__RefHeading___Toc25880)

[Figure **S11**. ^13^C NMR spectrum of compound **3b** 8](#__RefHeading___Toc18166)

[Figure **S12**. HRMS spectrum of compound **3b** 9](#__RefHeading___Toc31663)

[Figure **S13**. IR spectrum of compound **3c** 9](#__RefHeading___Toc26551)

[Figure **S14**. ^1^H NMR spectrum of compound **3c** 10](#__RefHeading___Toc31096)

[Figure **S15**. ^13^C NMR spectrum of compound **3c** 10](#__RefHeading___Toc9562)

[Figure **S16**. HRMS spectrum of compound **3c** 11](#__RefHeading___Toc5685)

[Figure **S17**. IR spectrum of compound **3d** 11](#__RefHeading___Toc24146)

[Figure **S18**. ^1^H NMR spectrum of compound **3d** 12](#__RefHeading___Toc3421)

[Figure **S19**. ^13^C NMR spectrum of compound **3d** 12](#__RefHeading___Toc9156)

[Figure **S20**. HRMS spectrum of compound **3d** 13](#__RefHeading___Toc2279)

[Figure **S21**. IR spectrum of compound **3e** 13](#__RefHeading___Toc26536)

[Figure **S22**. ^1^H NMR spectrum of compound **3e** 14](#__RefHeading___Toc19293)

[Figure **S23**. ^13^C NMR spectrum of compound **3e** 14](#__RefHeading___Toc24835)

[Figure **S24**. HRMS spectrum of compound **3e** 15](#__RefHeading___Toc23470)

[Figure **S25**. IR spectrum of compound **3f** 15](#__RefHeading___Toc32522)

[Figure **S26**. ^1^H NMR spectrum of compound **3f** 16](#__RefHeading___Toc18510)

[Figure **S27**. ^13^C NMR spectrum of compound **3f** 16](#__RefHeading___Toc18981)

[Figure **S28**. HRMS spectrum of compound **3f** 17](#__RefHeading___Toc1196)

[Figure **S29**. IR spectrum of compound **3g** 17](#__RefHeading___Toc18978)

[Figure **S30**. ^1^H NMR spectrum of compound **3g** 18](#__RefHeading___Toc15112)

[Figure **S31**. ^13^C NMR spectrum of compound **3g** 18](#__RefHeading___Toc815)

[Figure **S32**. HRMS spectrum of compound **3g** 19](#__RefHeading___Toc32291)

[Figure **S33**. IR spectrum of compound **3h** 19](#__RefHeading___Toc26432)

[Figure **S34**. ^1^H NMR spectrum of compound **3h** 20](#__RefHeading___Toc24940)

[Figure **S35**. ^13^C NMR spectrum of compound **3h** 20](#__RefHeading___Toc18449)

[Figure **S36**. HRMS spectrum of compound **3h** 21](#__RefHeading___Toc29587)

[Figure **S37**. IR spectrum of compound **3i** 21](#__RefHeading___Toc30190)

[Figure **S38**. ^1^H NMR spectrum of compound **3i** 22](#__RefHeading___Toc13990)

[Figure **S39**. ^13^C NMR spectrum of compound **3i** 22](#__RefHeading___Toc23000)

[Figure **S40**. HRMS spectrum of compound **3i** 23](#__RefHeading___Toc18979)

[Figure **S41**. IR spectrum of compound **3j** 23](#__RefHeading___Toc3530)

[Figure **S42**. ^1^H NMR spectrum of compound **3j** 24](#__RefHeading___Toc24238)

[Figure **S43**. ^13^C NMR spectrum of compound **3j** 24](#__RefHeading___Toc19494)

[Figure **S44**. HRMS spectrum of compound **3j** 25](#__RefHeading___Toc2114)

[Figure **S45**. IR spectrum of compound **3k** 25](#__RefHeading___Toc6037)

[Figure **S46**. ^1^H NMR spectrum of compound **3k** 26](#__RefHeading___Toc27413)

[Figure **S47**. ^13^C NMR spectrum of compound **3k** 26](#__RefHeading___Toc18143)

[Figure **S48**. HRMS spectrum of compound **3k** 27](#__RefHeading___Toc24718)

[Figure **S49**. IR spectrum of compound **3l** 27](#__RefHeading___Toc15495)

[Figure **S50**. ^1^H NMR spectrum of compound **3l** 28](#__RefHeading___Toc23806)

[Figure **S51**. ^13^C NMR spectrum of compound **3l** 28](#__RefHeading___Toc4134)

[Figure **S52**. HRMS spectrum of compound **3l** 29](#__RefHeading___Toc25589)

[Figure **S53**. IR spectrum of compound **3m** 29](#__RefHeading___Toc24549)

[Figure **S54**. ^1^H NMR spectrum of compound **3m** 30](#__RefHeading___Toc2155)

[Figure **S55**. ^13^C NMR spectrum of compound **3m** 30](#__RefHeading___Toc29320)

[Figure **S56**. HRMS spectrum of compound **3m** 31](#__RefHeading___Toc7962)

[Figure **S57**. IR spectrum of compound **3n** 31](#__RefHeading___Toc22230)

[Figure **S58**. ^1^H NMR spectrum of compound **3n** 32](#__RefHeading___Toc15546)

[Figure **S59**. ^13^C NMR spectrum of compound **3n** 32](#__RefHeading___Toc21366)

[Figure **S60**. HRMS spectrum of compound **3n** 33](#__RefHeading___Toc9978)

[Figure **S61**. IR spectrum of compound **3o** 33](#__RefHeading___Toc21107)

[Figure **S62**. ^1^H NMR spectrum of compound **3o** 34](#__RefHeading___Toc22127)

[Figure **S63**. ^13^C NMR spectrum of compound **3o** 34](#__RefHeading___Toc3577)

[Figure **S64**. HRMS spectrum of compound **3o** 35](#__RefHeading___Toc22735)

[Figure **S65**. IR spectrum of compound **3p** 35](#__RefHeading___Toc12317)

[Figure **S66**. ^1^H NMR spectrum of compound **3p** 36](#__RefHeading___Toc7731)

[Figure **S67**. ^13^C NMR spectrum of compound **3p** 36](#__RefHeading___Toc26017)

[Figure **S68**. HRMS spectrum of compound **3p** 37](#__RefHeading___Toc20789)

Figure **S1**. IR spectrum of compound **2**


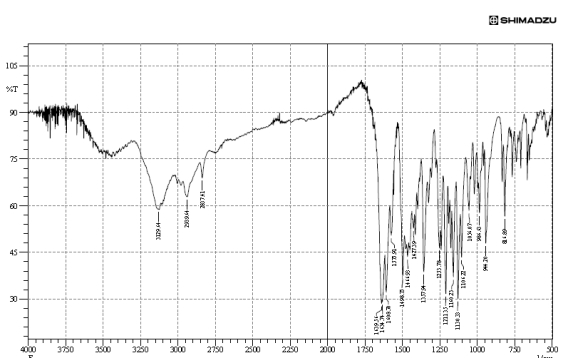


Figure **S2**. ^1^H NMR spectrum of compound **2**

**
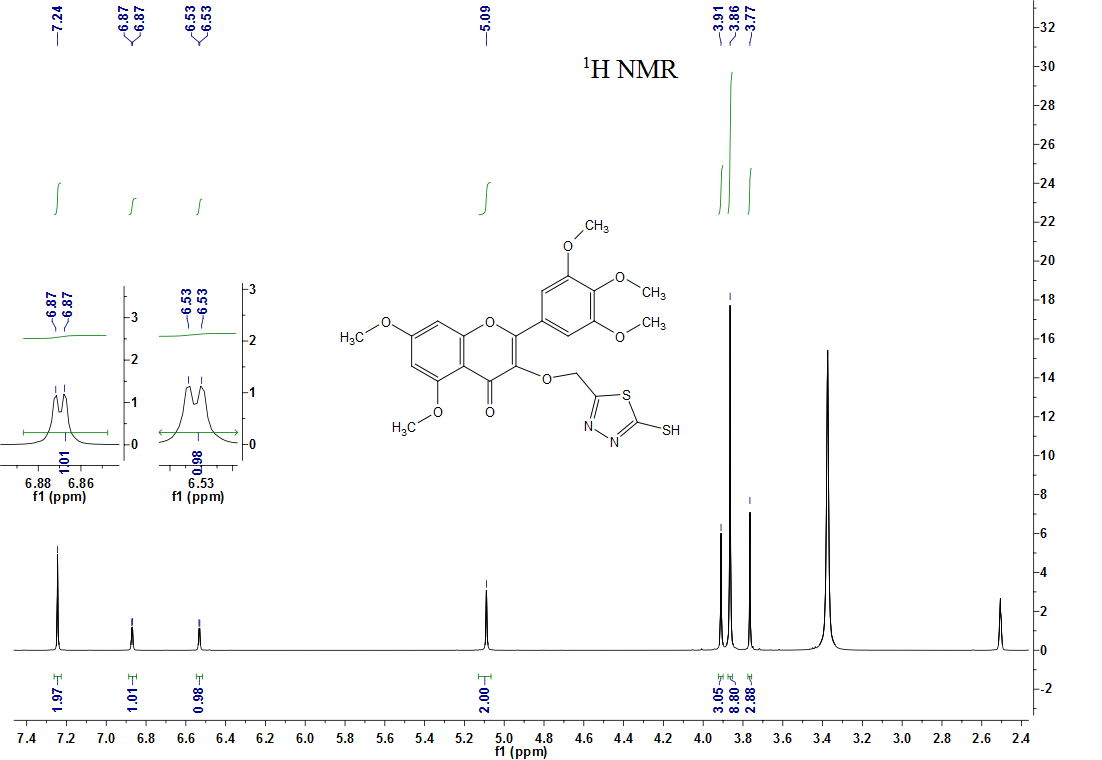
**

Figure **S3**. ^13^C NMR spectrum of compound **2**

**
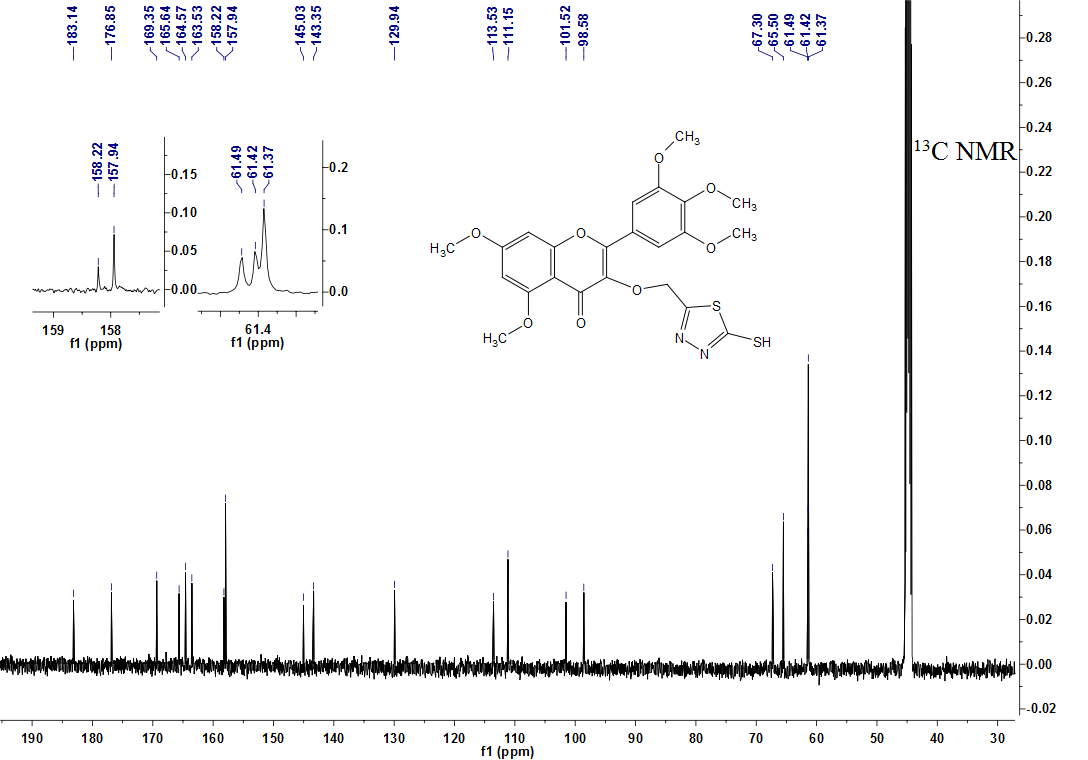
**

Figure **S4**. HRMS spectrum of compound **2**

Figure **S5**. IR spectrum of compound **3a**


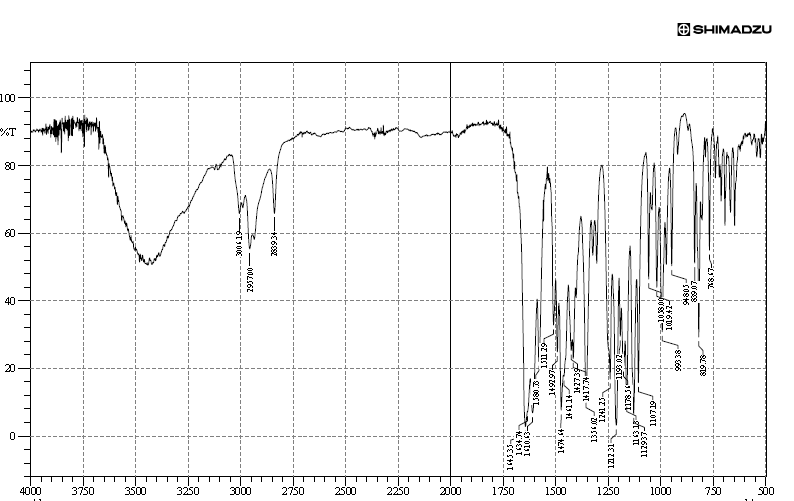


Figure **S6**. ^1^H NMR spectrum of compound **3a**

**
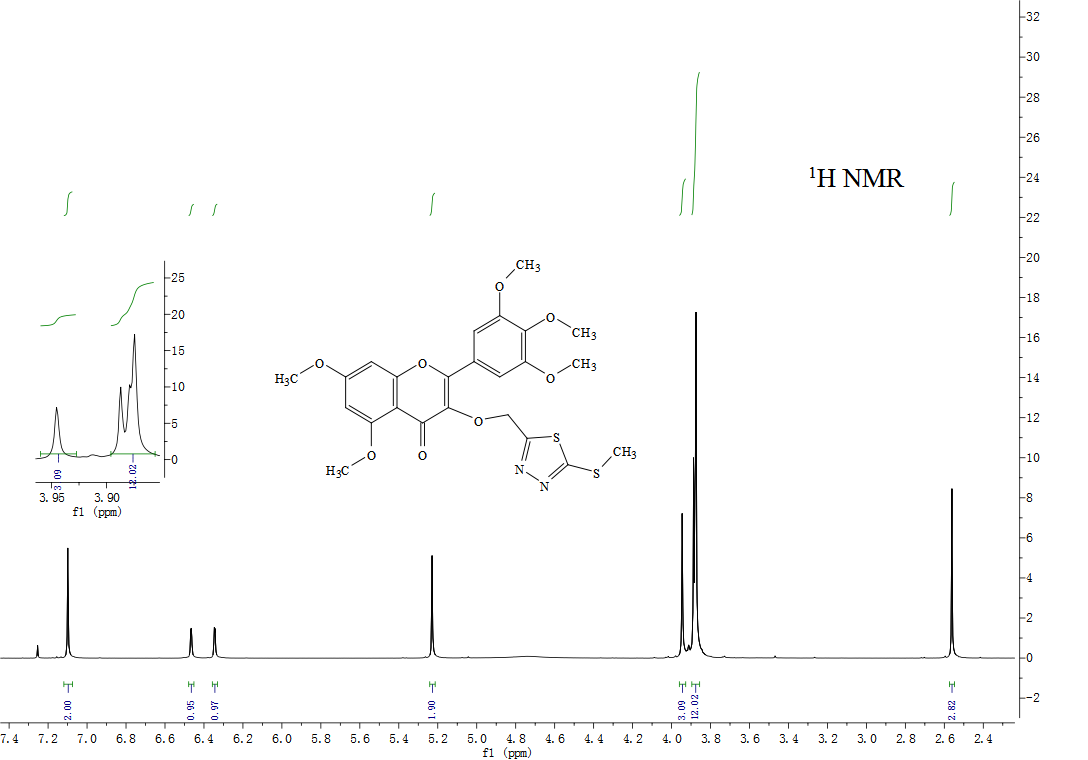
**

Figure **S7**. ^13^C NMR spectrum of compound **3a**

**
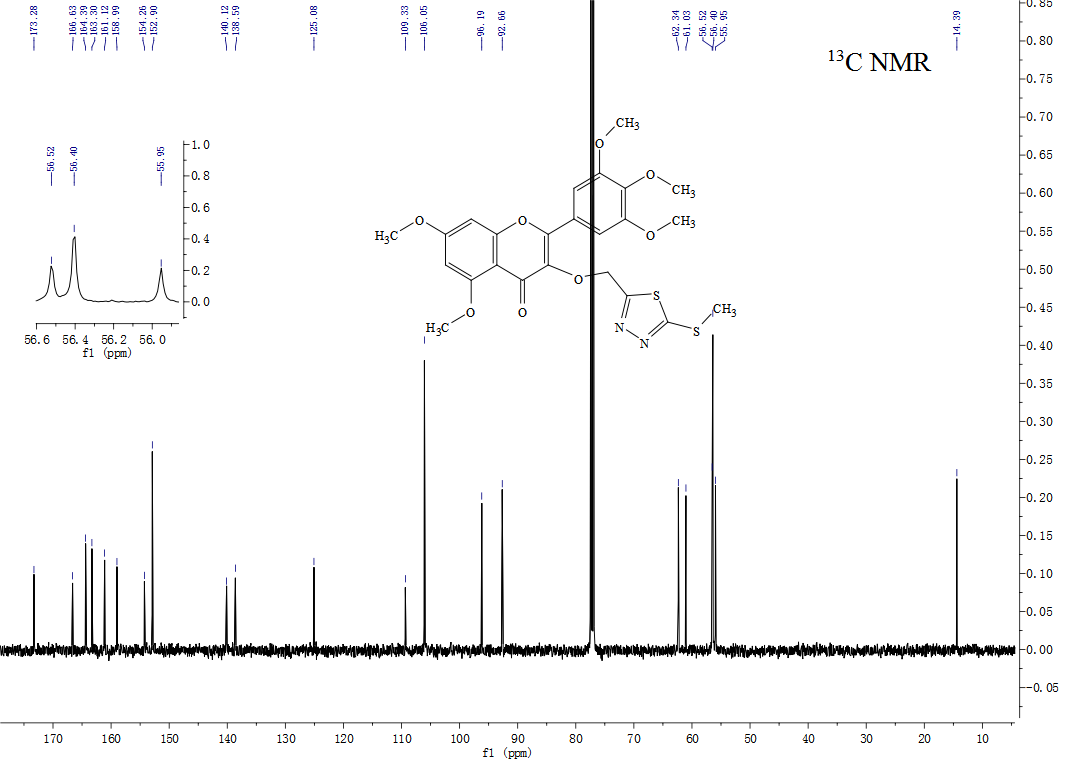
**

Figure **S8**. HRMS spectrum of compound **3a**

Figure **S9**. IR spectrum of compound **3b**


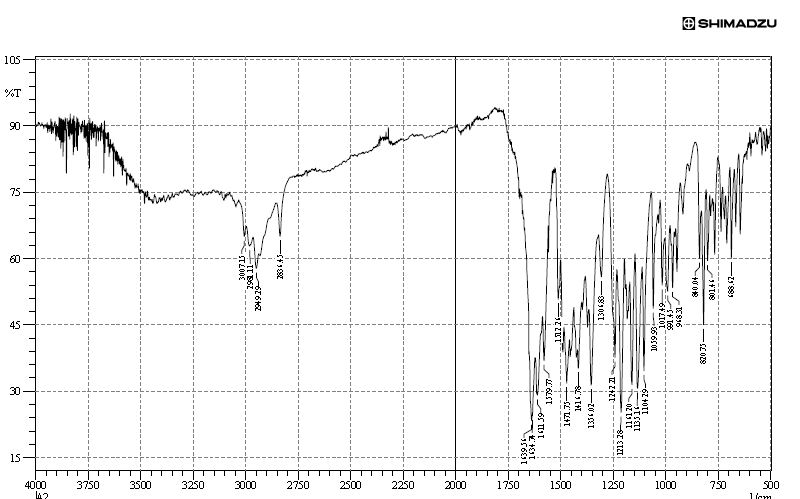


Figure **S10**. ^1^H NMR spectrum of compound **3b**

**
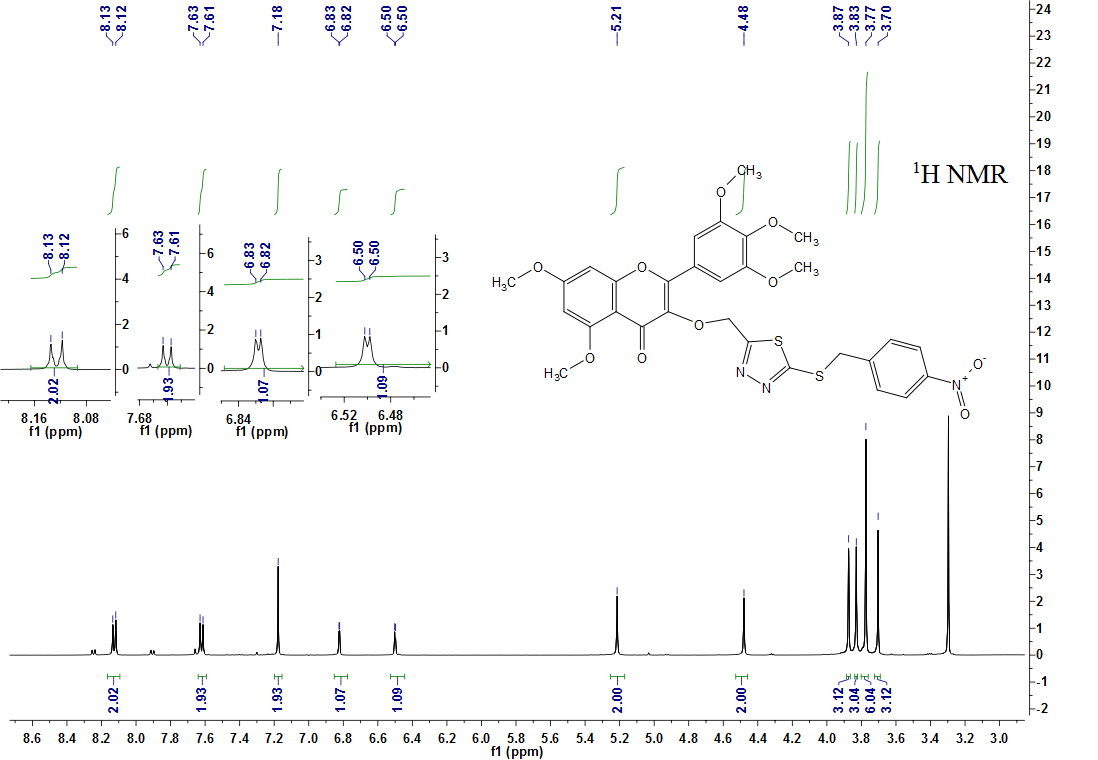
**

Figure **S11**. ^13^C NMR spectrum of compound **3b**

**
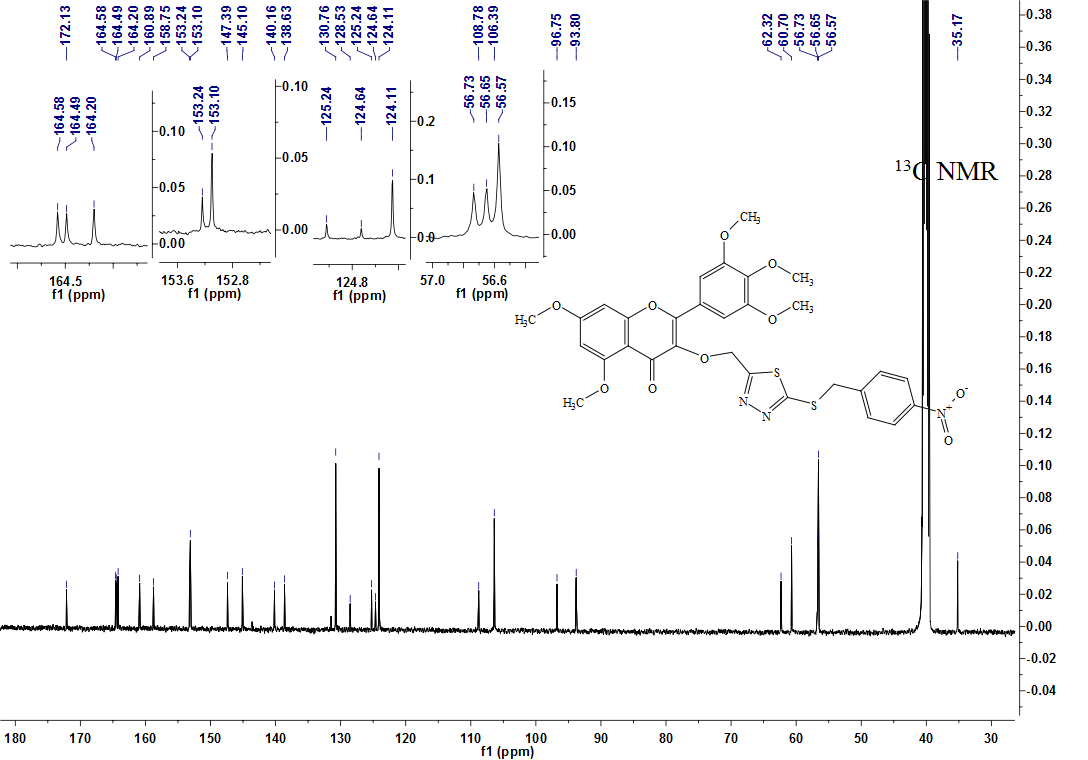
**

Figure **S12**. HRMS spectrum of compound **3b**

Figure **S13**. IR spectrum of compound **3c**


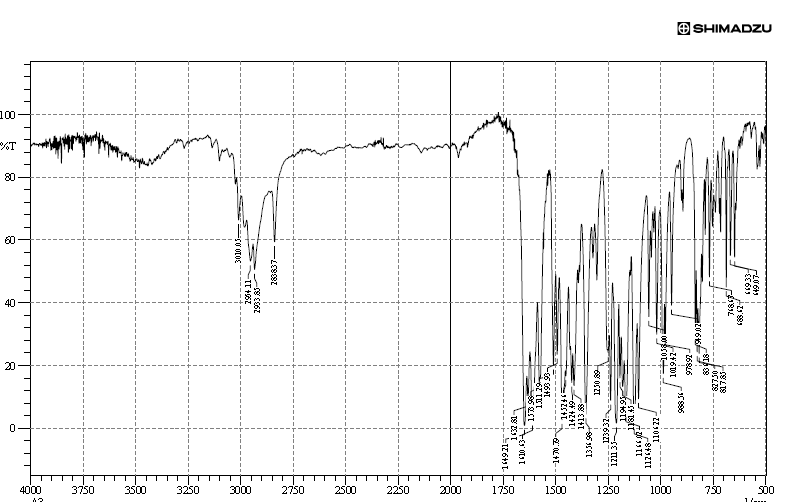


Figure **S14**. ^1^H NMR spectrum of compound **3c**

**
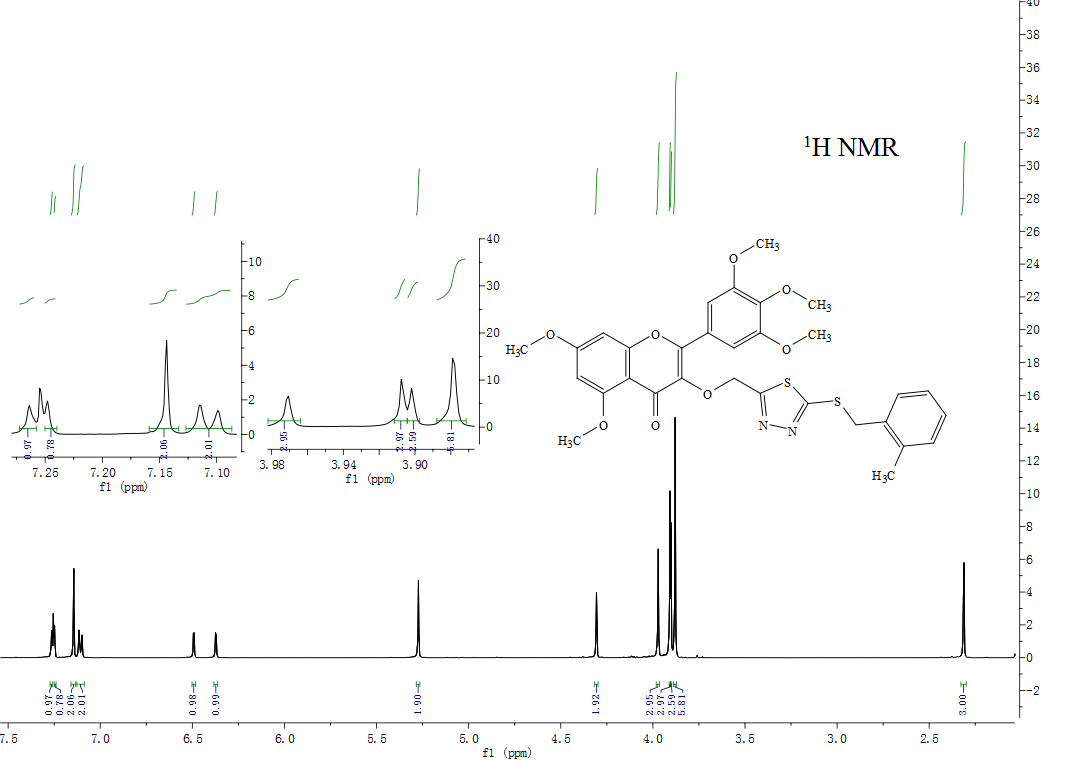
**

Figure **S15**. ^13^C NMR spectrum of compound **3c**

**
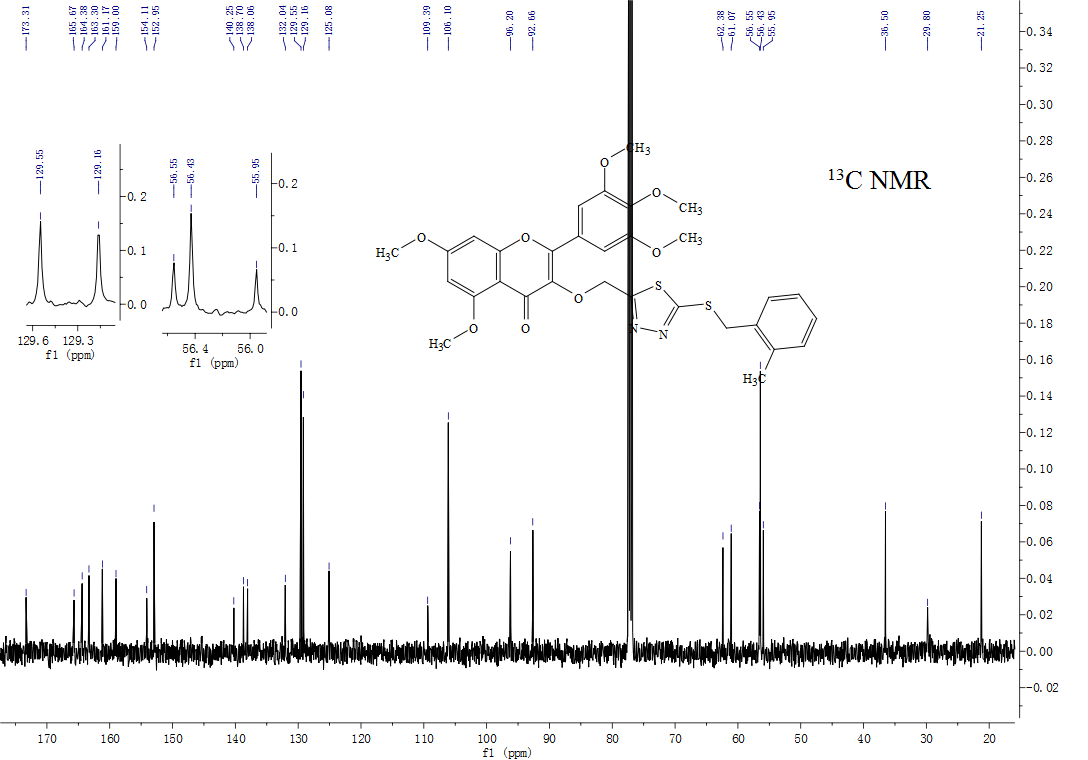
**

Figure **S16**. HRMS spectrum of compound **3c**

Figure **S17**. IR spectrum of compound **3d**


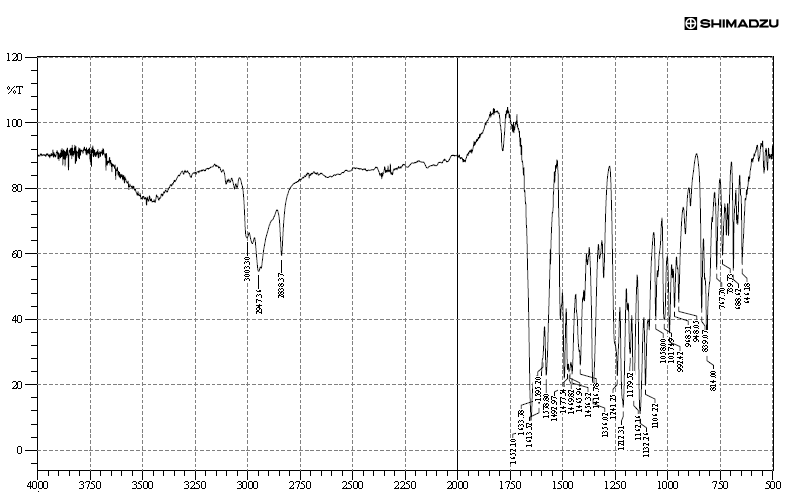


Figure **S18**. ^1^H NMR spectrum of compound **3d**

**
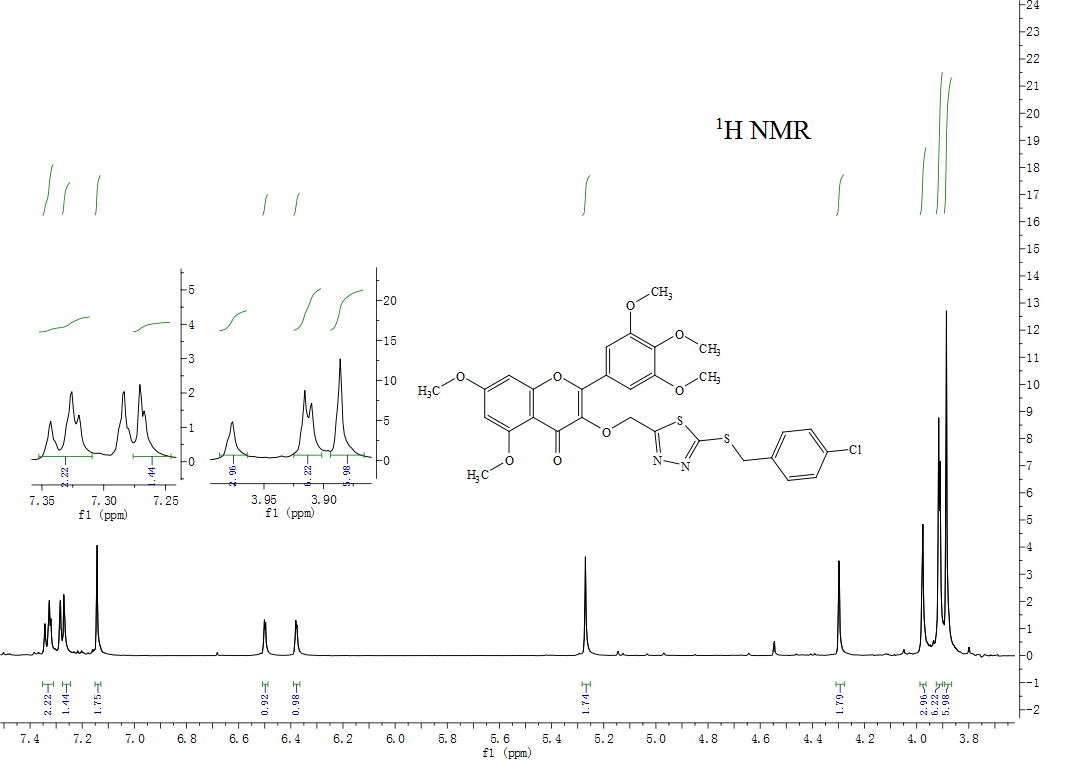
**

Figure **S19**. ^13^C NMR spectrum of compound **3d**

**
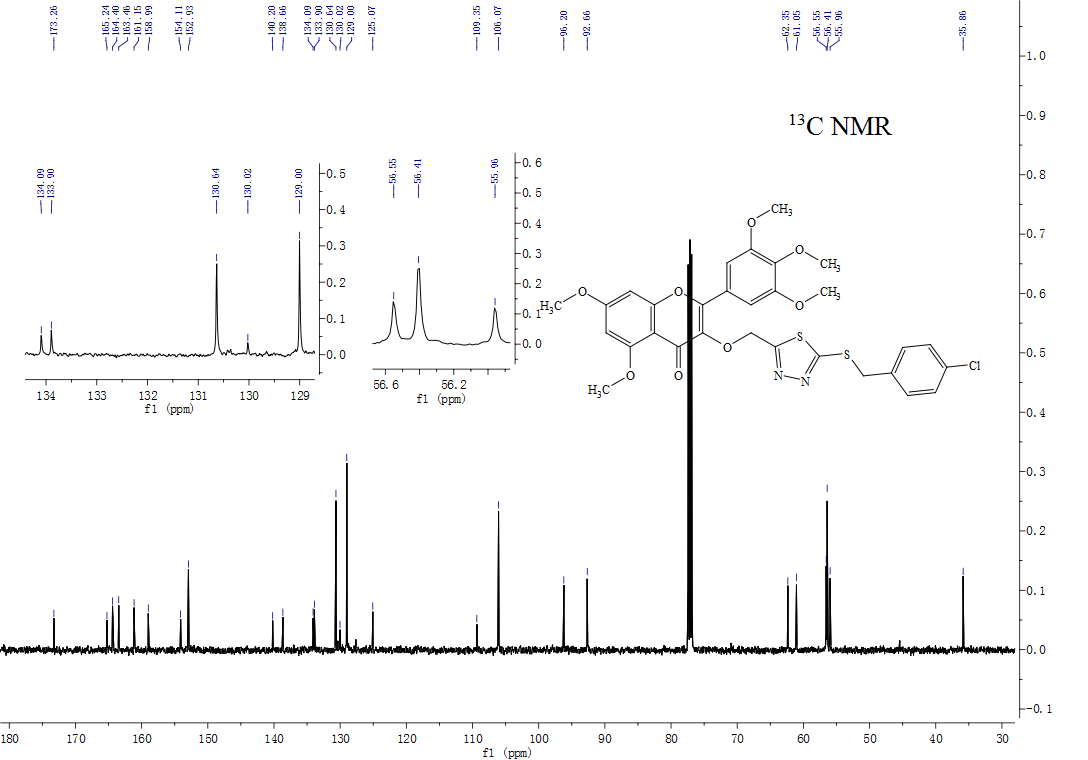
**

Figure **S20**. HRMS spectrum of compound **3d**

Figure **S21**. IR spectrum of compound **3e**


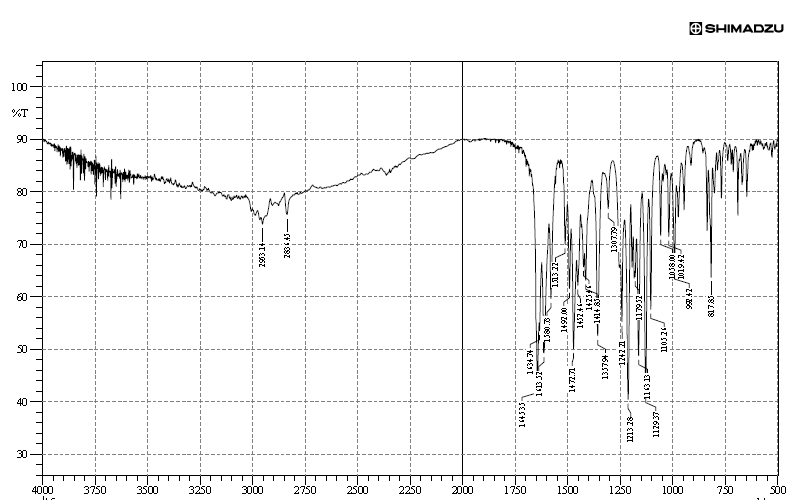


Figure **S22**. ^1^H NMR spectrum of compound **3e**

**
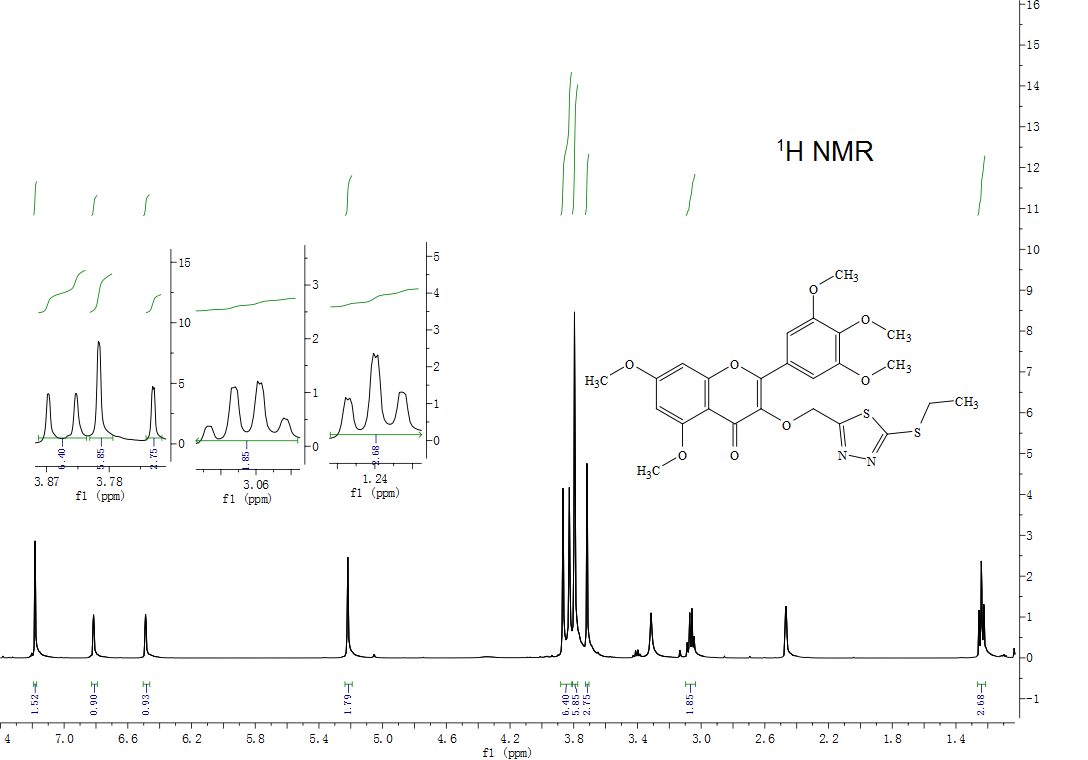
**

Figure **S23**. ^13^C NMR spectrum of compound **3e**

**
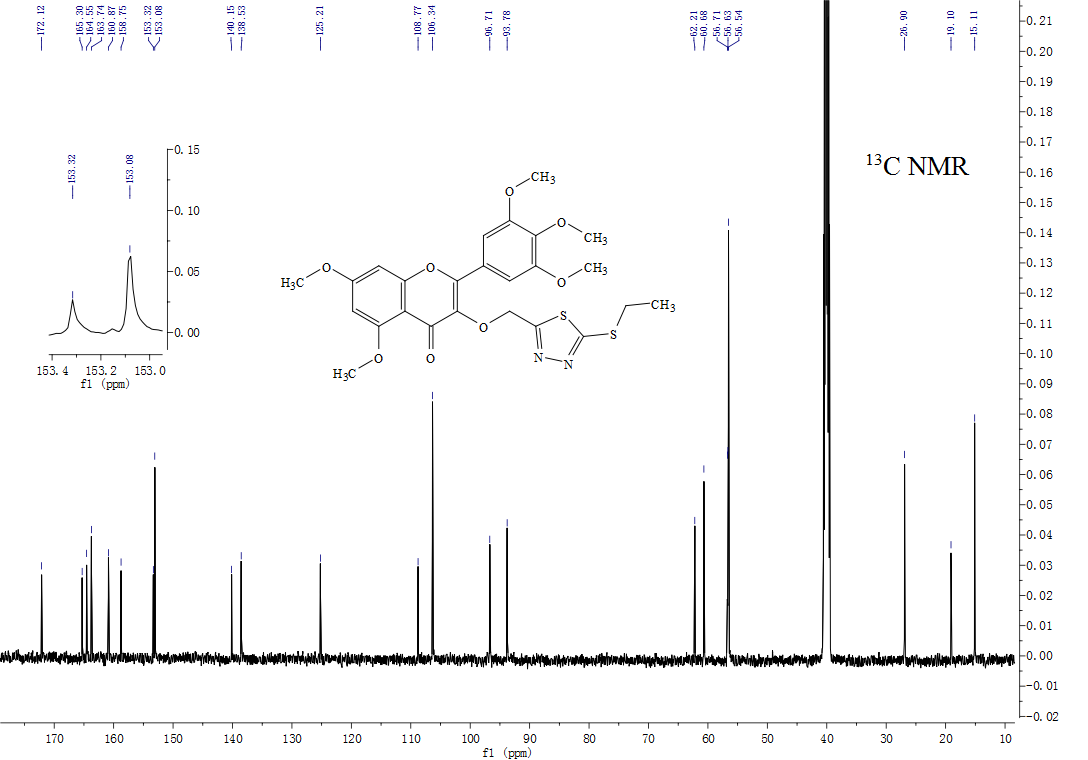
**

Figure **S24**. HRMS spectrum of compound **3e**

Figure **S25**. IR spectrum of compound **3f**


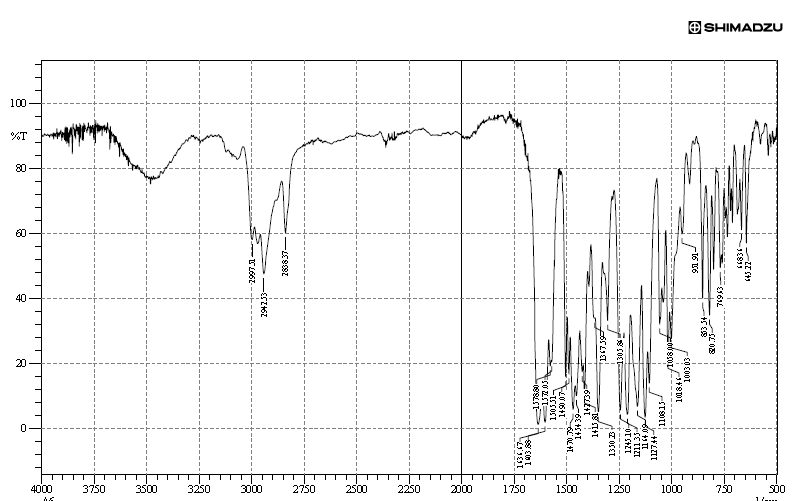


Figure **S26**. ^1^H NMR spectrum of compound **3f**

**
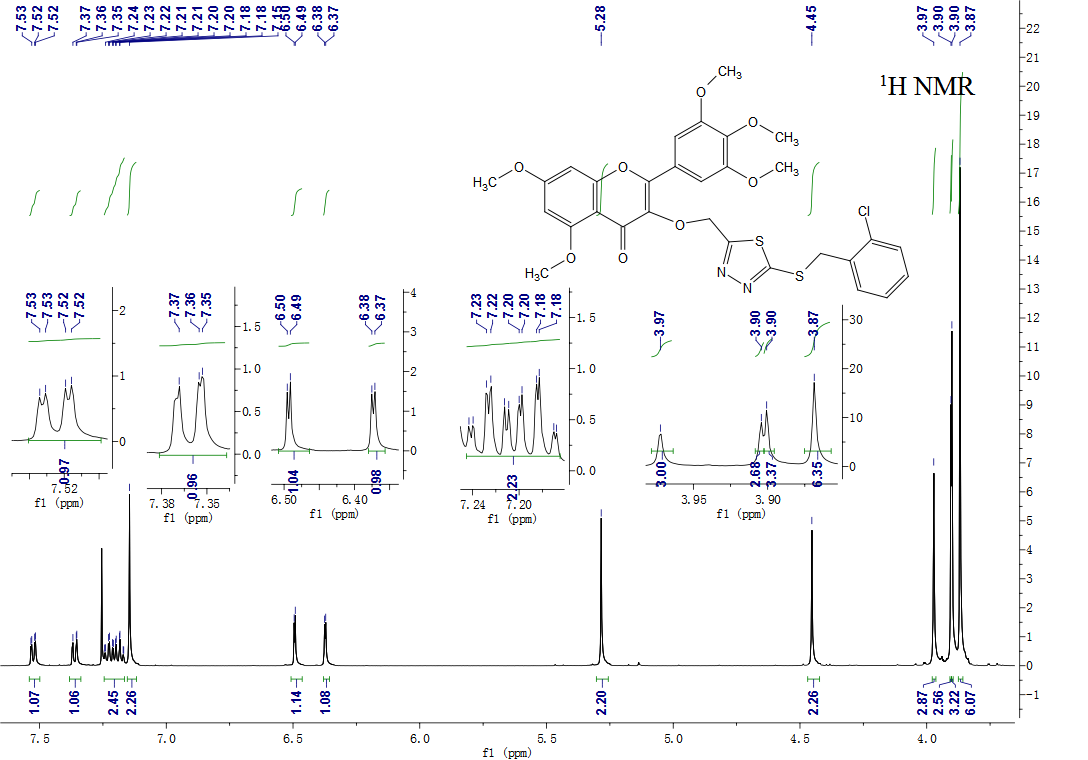
**

Figure **S27**. ^13^C NMR spectrum of compound **3f**

**
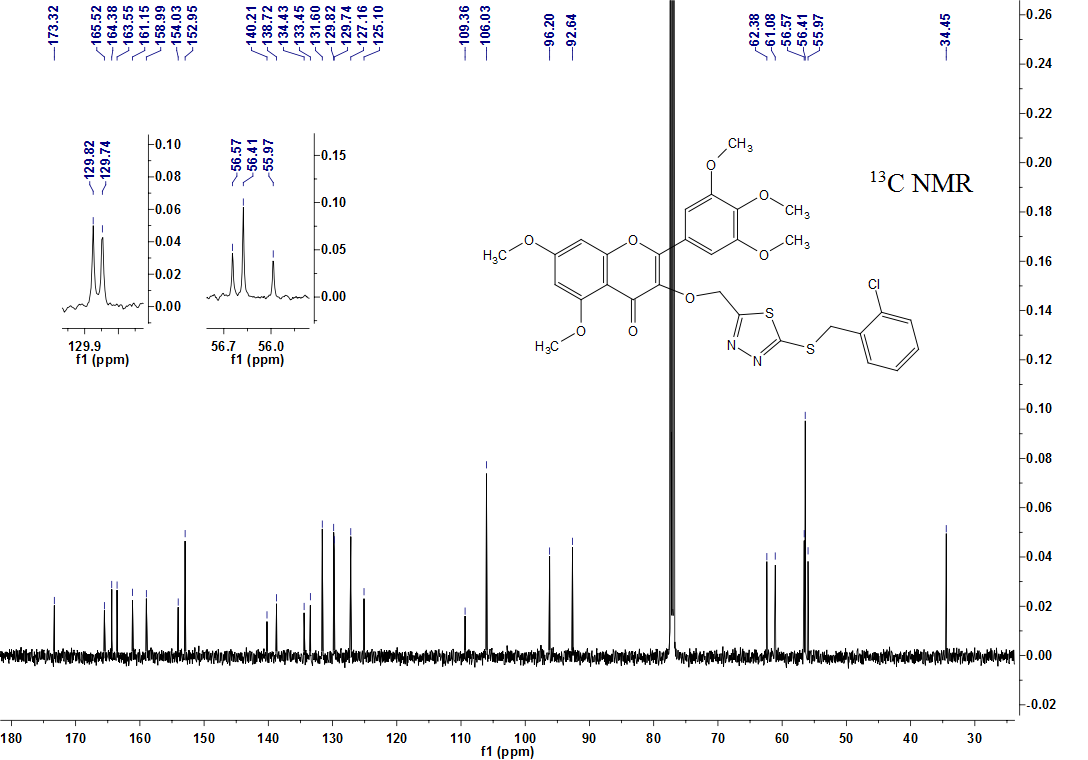
**

Figure **S28**. HRMS spectrum of compound **3f**

Figure **S29**. IR spectrum of compound **3g**


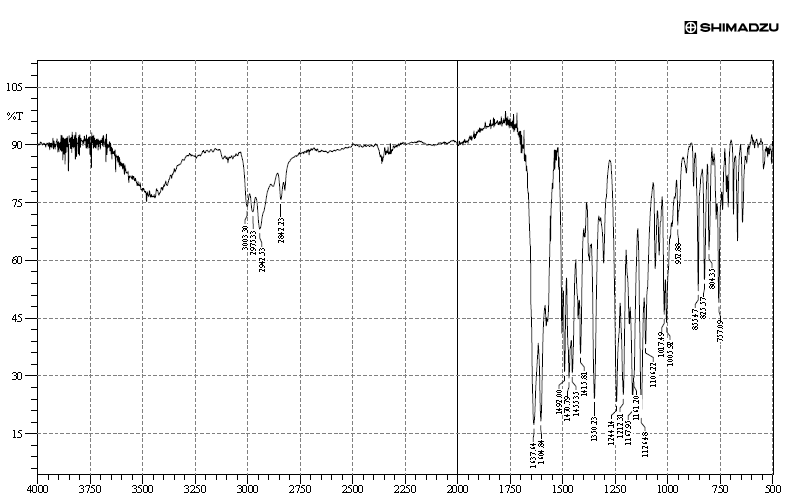


Figure **S30**. ^1^H NMR spectrum of compound **3g**

**
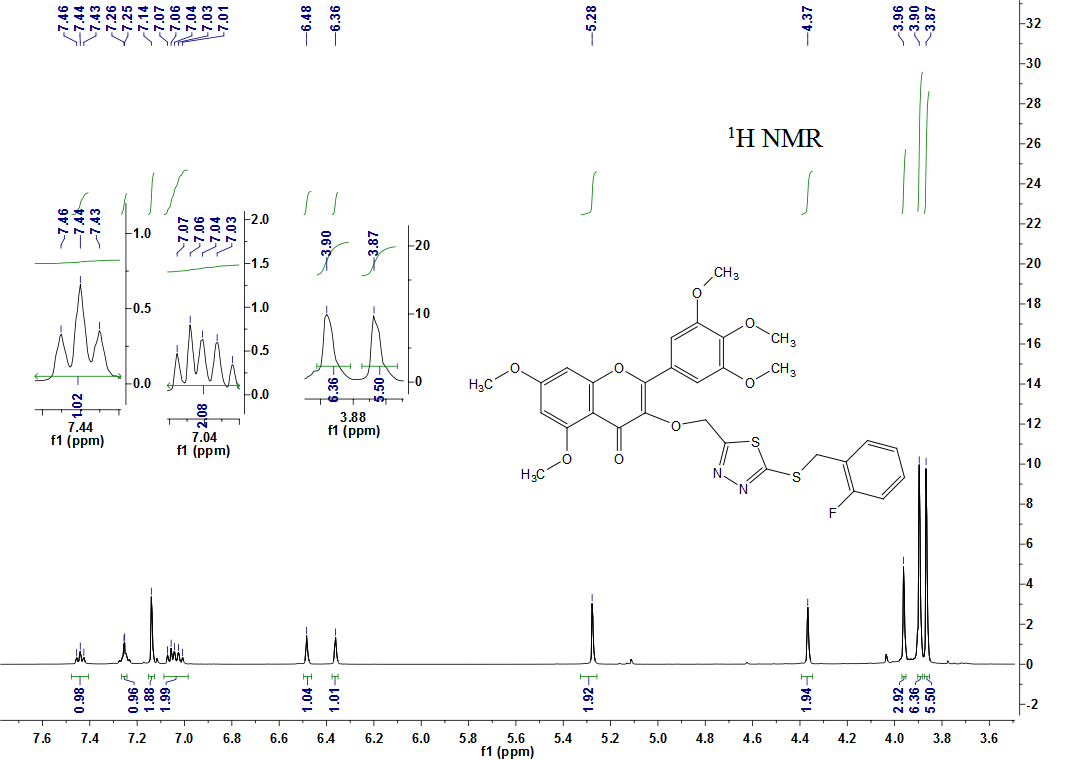
**

Figure **S31**. ^13^C NMR spectrum of compound **3g**

**
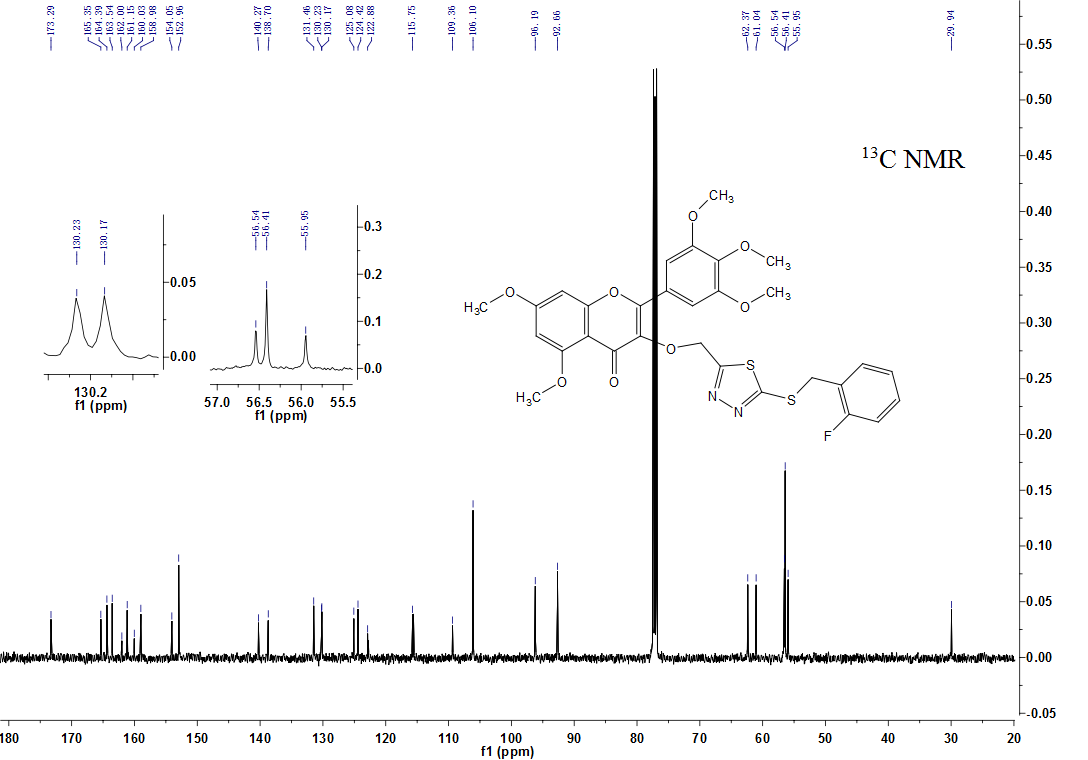
**

Figure **S32**. HRMS spectrum of compound **3g**

Figure **S33**. IR spectrum of compound **3h**


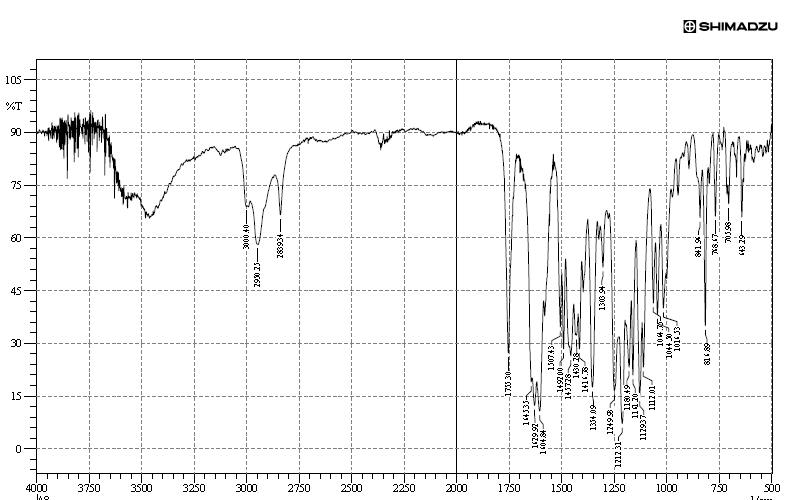


Figure **S34**. ^1^H NMR spectrum of compound **3h**

**
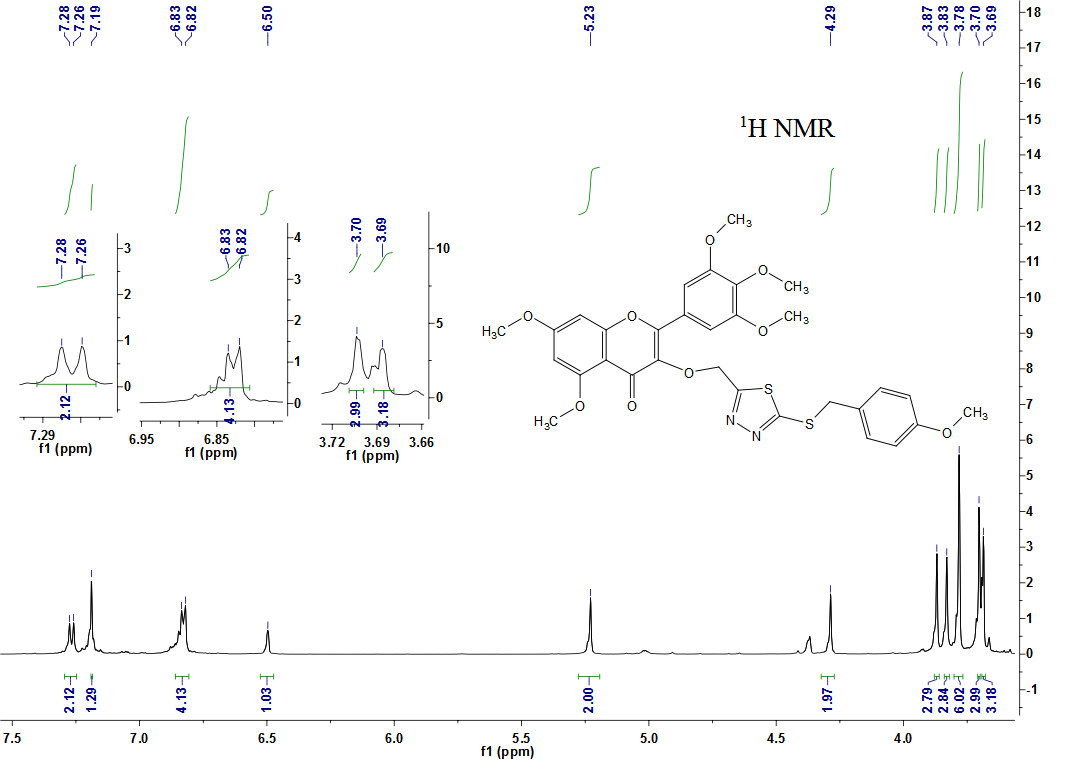
**

Figure **S35**. ^13^C NMR spectrum of compound **3h**

**
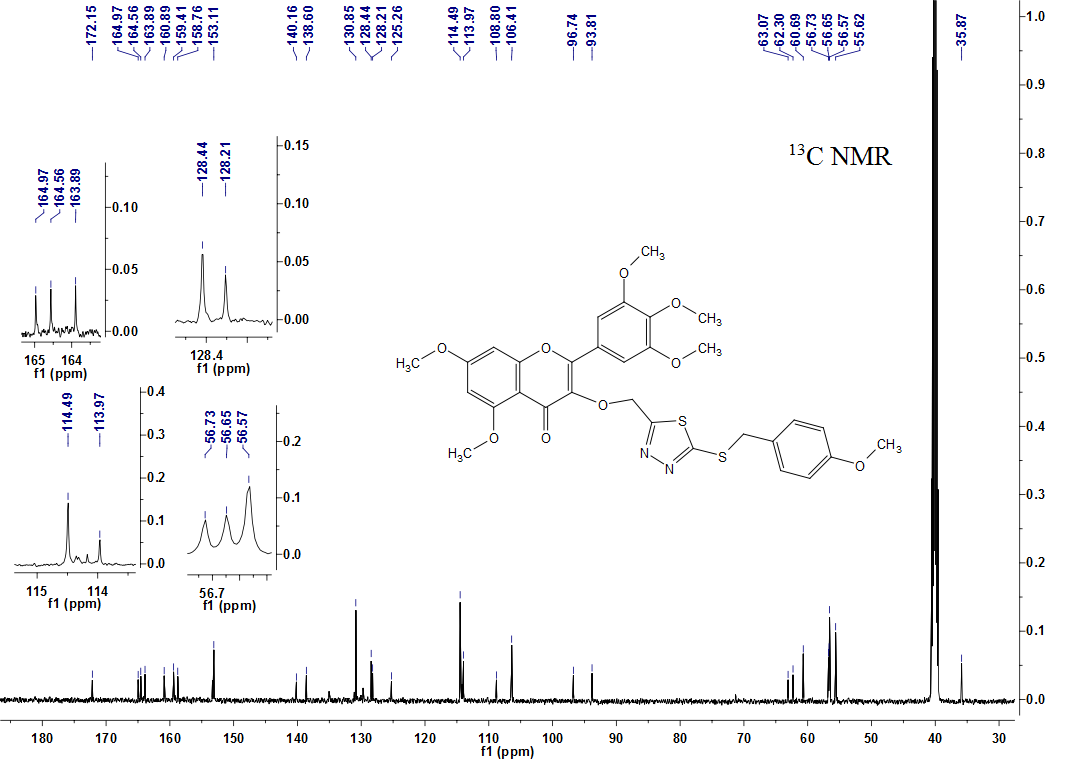
**

Figure **S36**. HRMS spectrum of compound **3h**

Figure **S37**. IR spectrum of compound **3i**


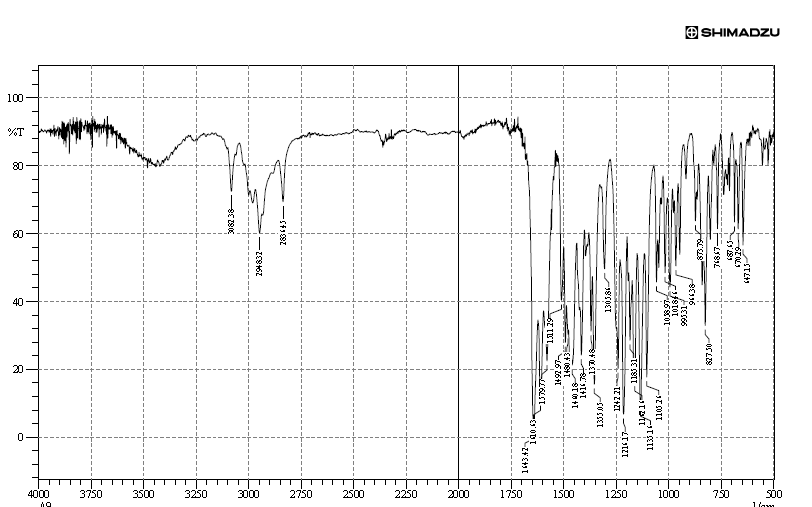


Figure **S38**. ^1^H NMR spectrum of compound **3i**

**
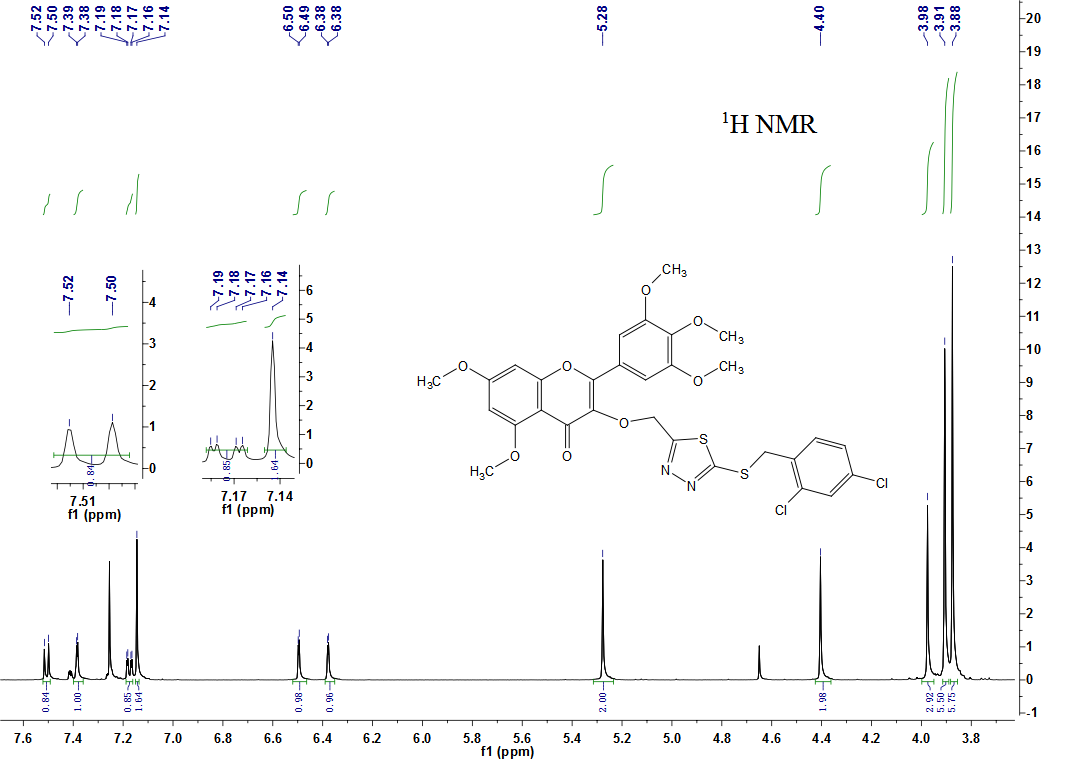
**

Figure **S39**. ^13^C NMR spectrum of compound **3i**

**
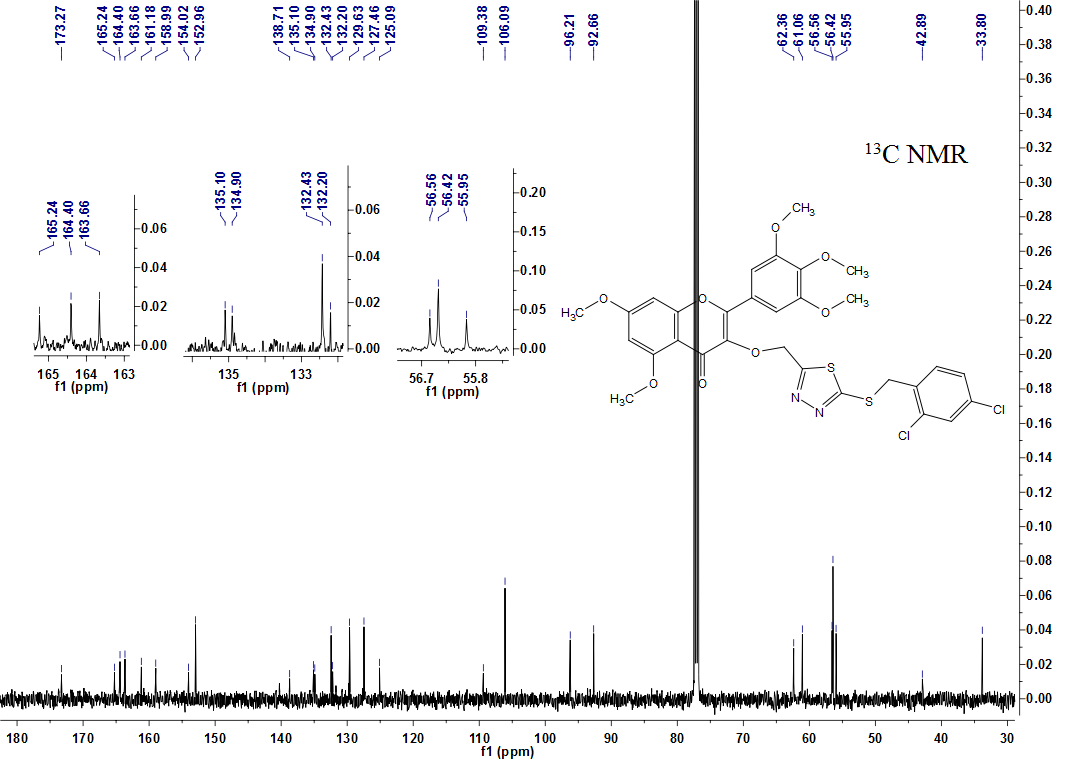
**

*Figure* ***S40****. HRMS spectrum of compound* ***3i***

Figure **S41**. IR spectrum of compound **3j**


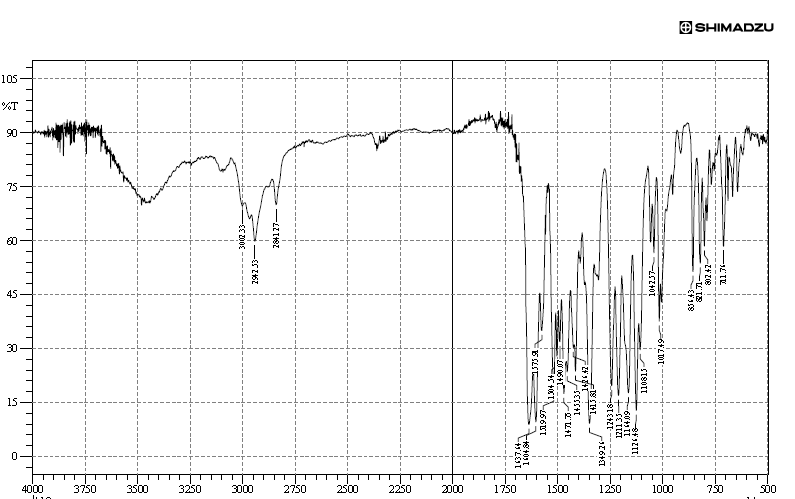


Figure **S42**. ^1^H NMR spectrum of compound **3j**

**
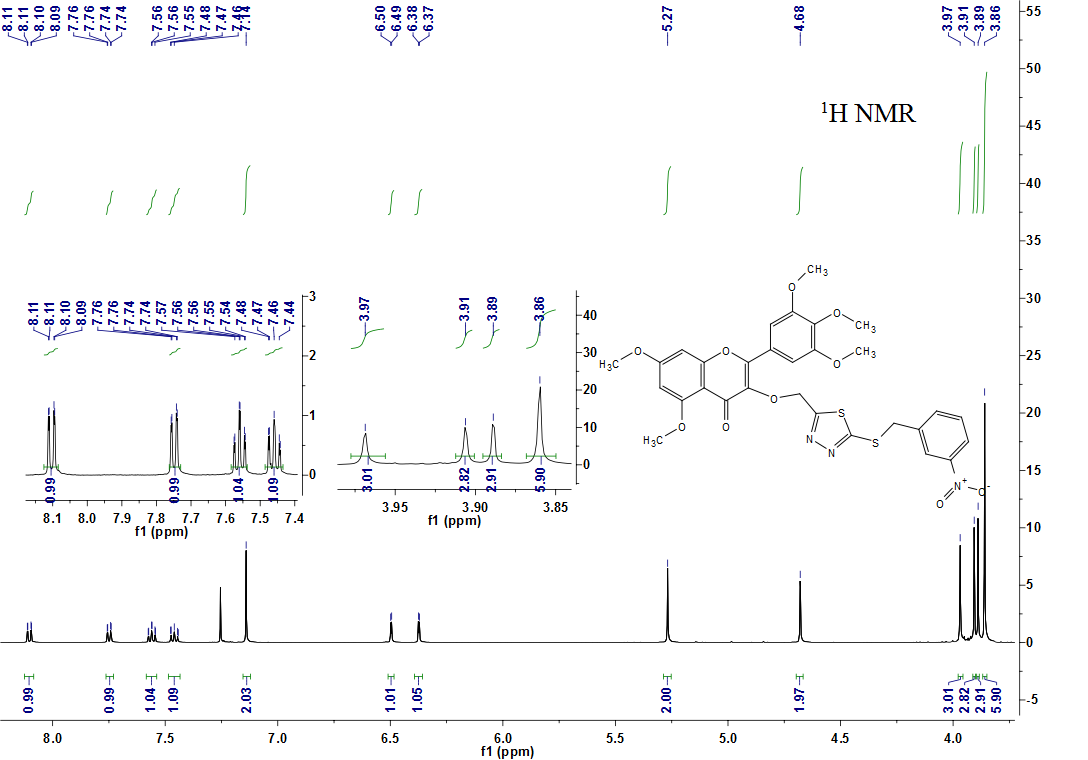
**

Figure **S43**. ^13^C NMR spectrum of compound **3j**

**
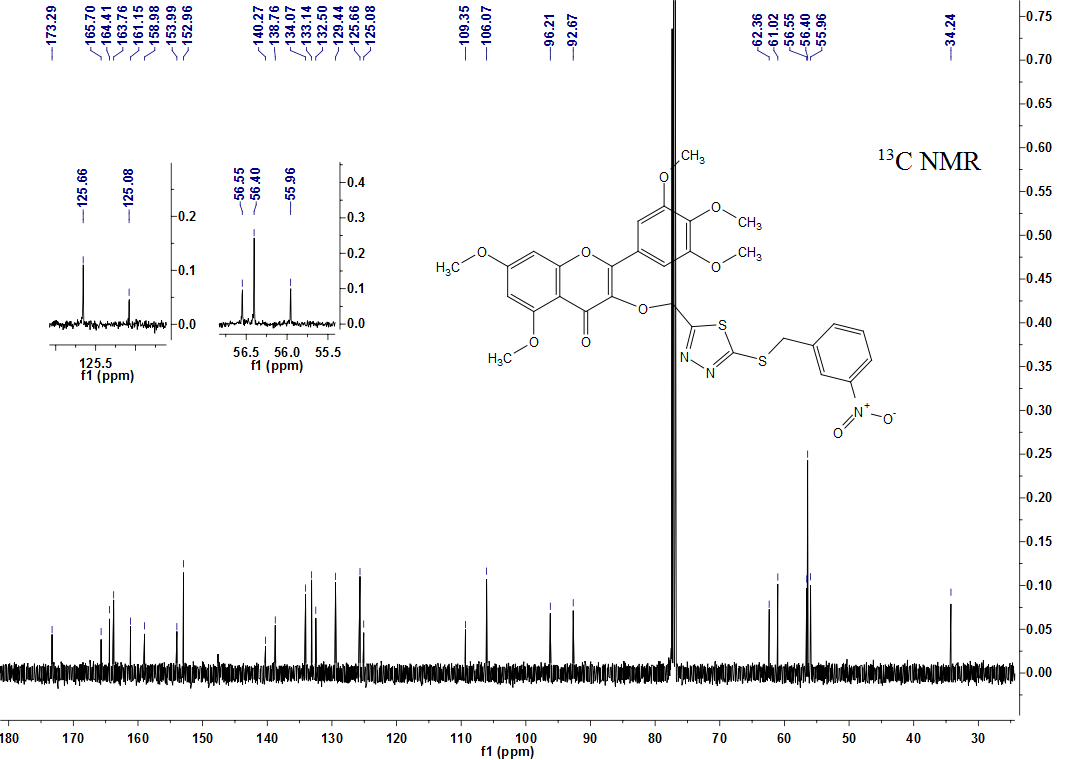
**

*Figure* ***S44****. HRMS spectrum of compound* ***3j***

Figure **S45**. IR spectrum of compound **3k**


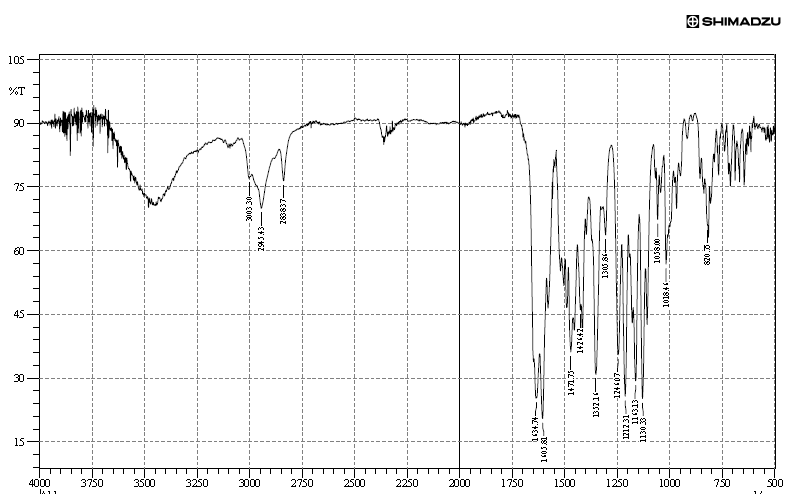


Figure **S46**. ^1^H NMR spectrum of compound **3k**

**
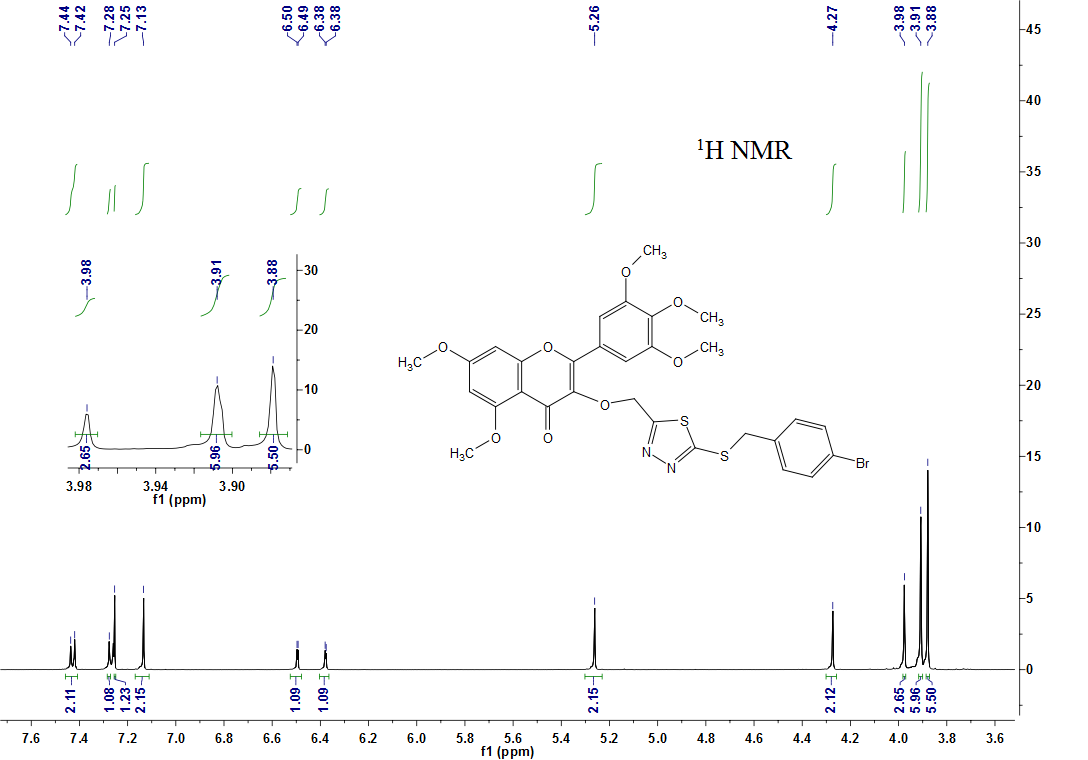
**

Figure **S47**. ^13^C NMR spectrum of compound **3k**

**
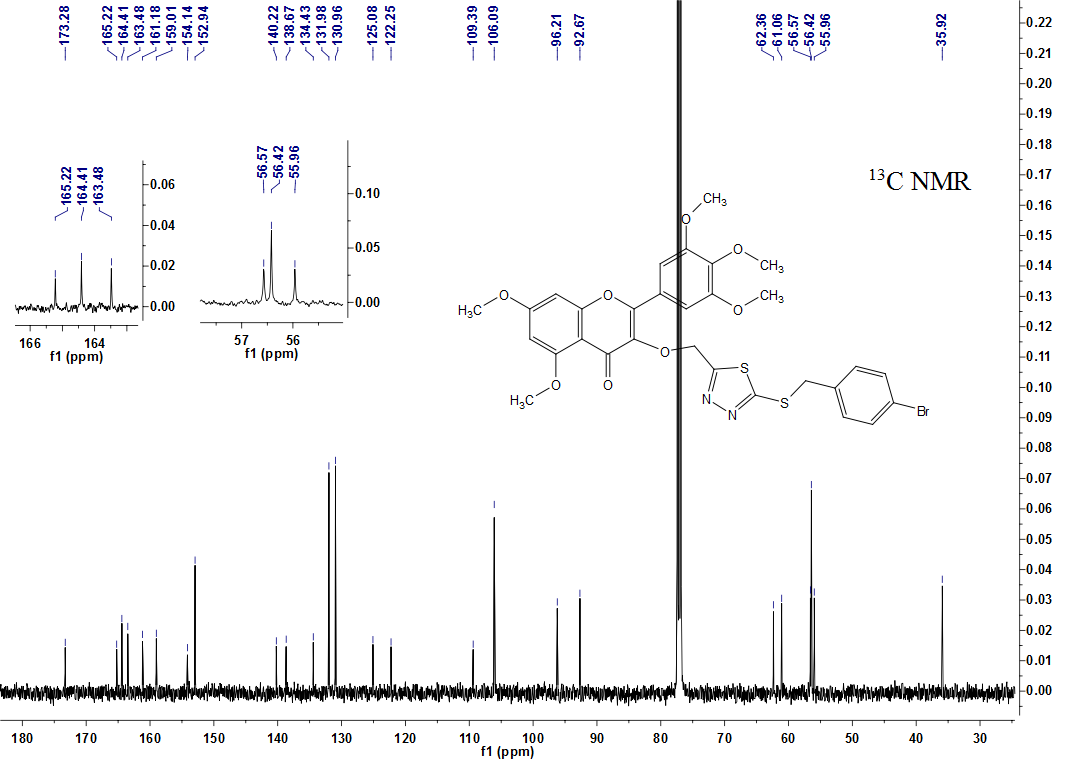
**

*Figure* ***S48****. HRMS spectrum of compound* ***3k***

Figure **S49**. IR spectrum of compound **3l**


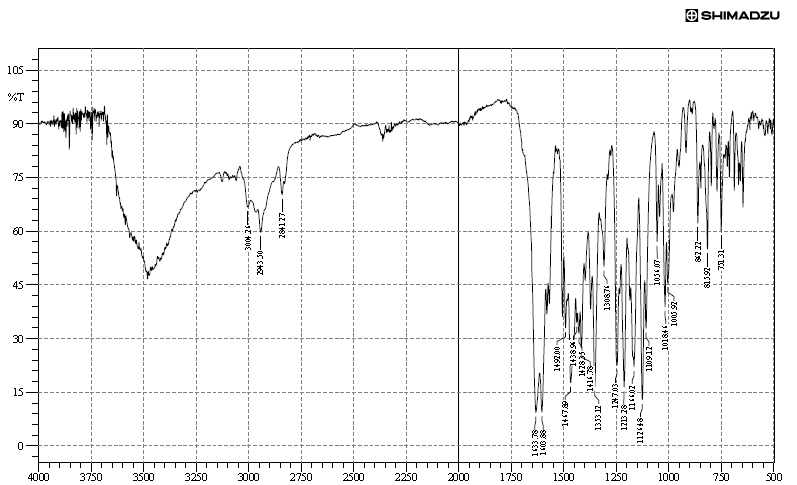


Figure **S50**. ^1^H NMR spectrum of compound **3l**

**
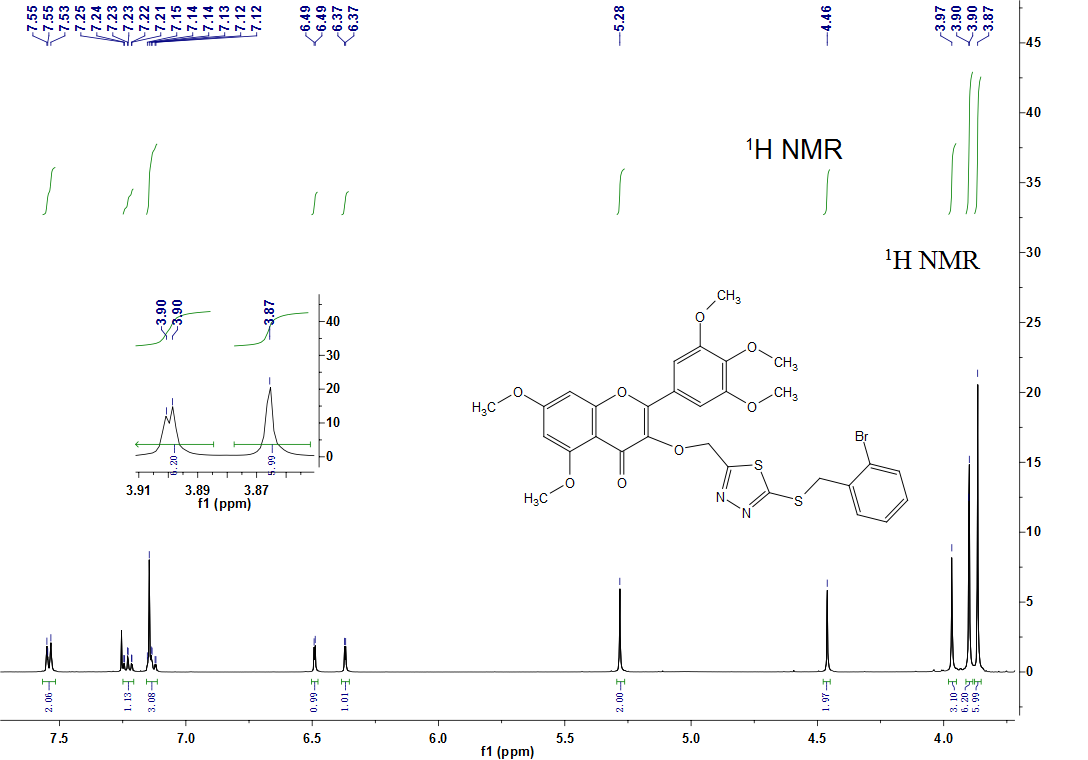
**

Figure **S51**. ^13^C NMR spectrum of compound **3l**

**
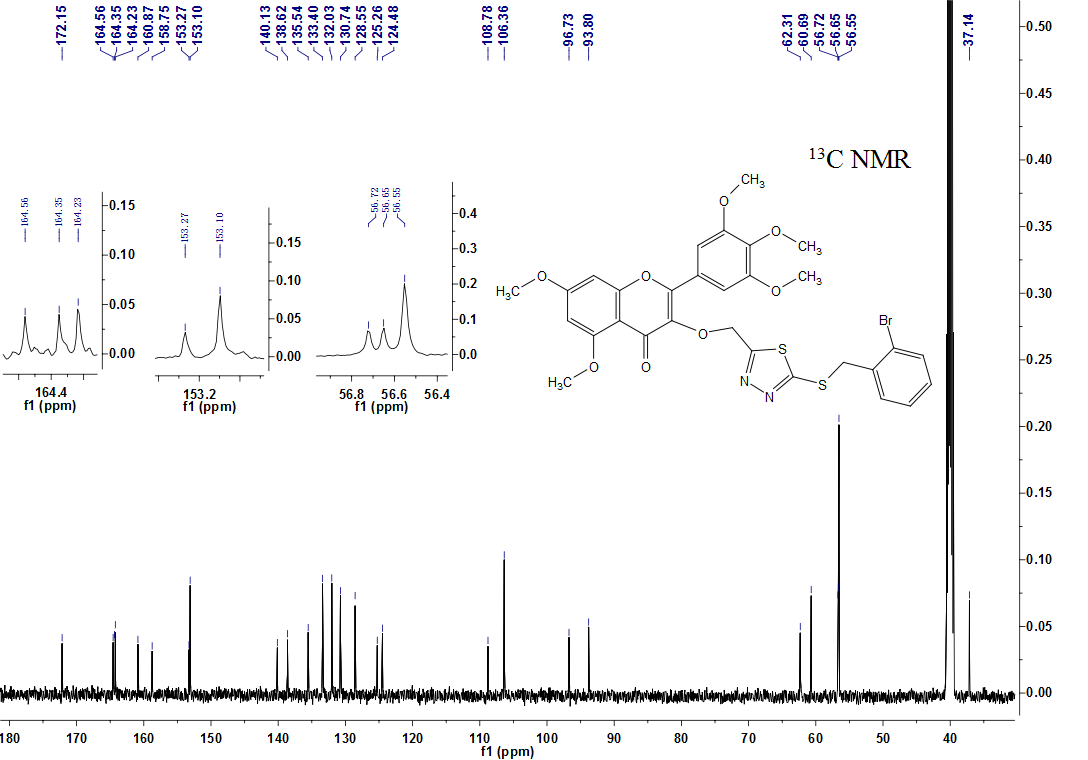
**

*Figure* ***S52****. HRMS spectrum of compound* ***3l***

Figure **S53**. IR spectrum of compound **3m**


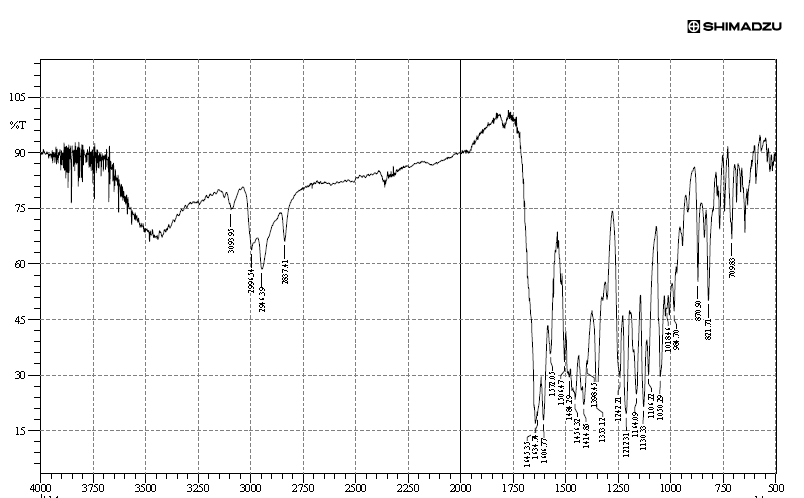


Figure **S54**. ^1^H NMR spectrum of compound **3m**

**
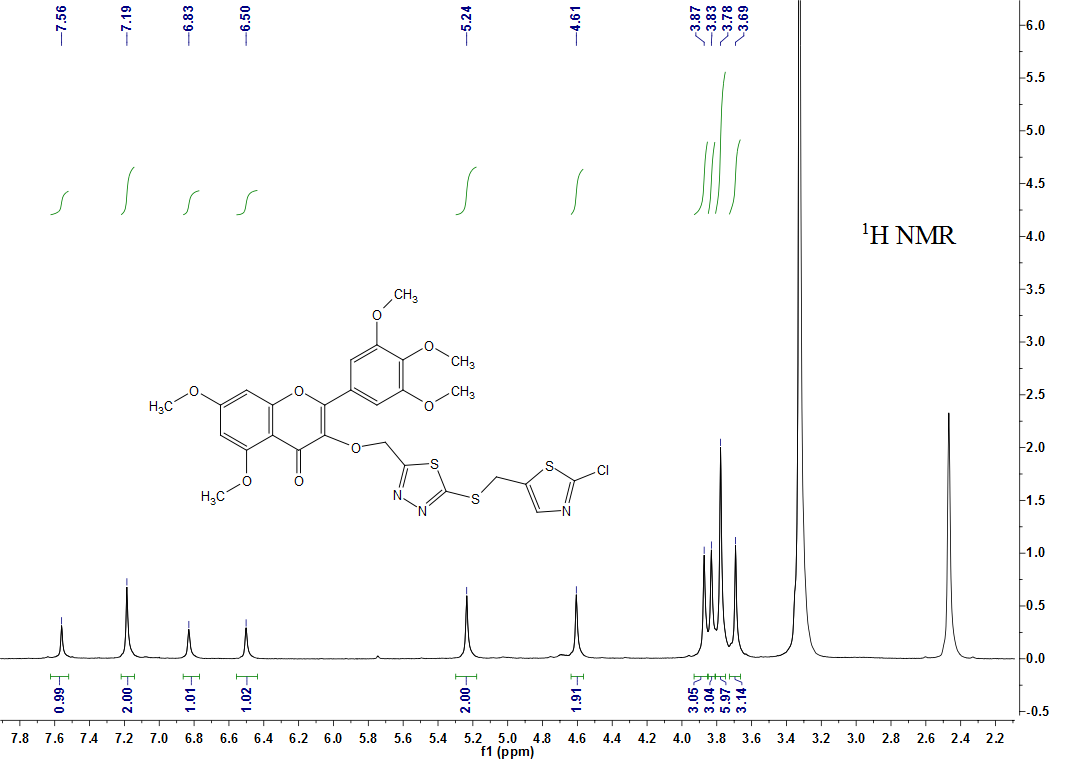
**

Figure **S55**. ^13^C NMR spectrum of compound **3m**

**
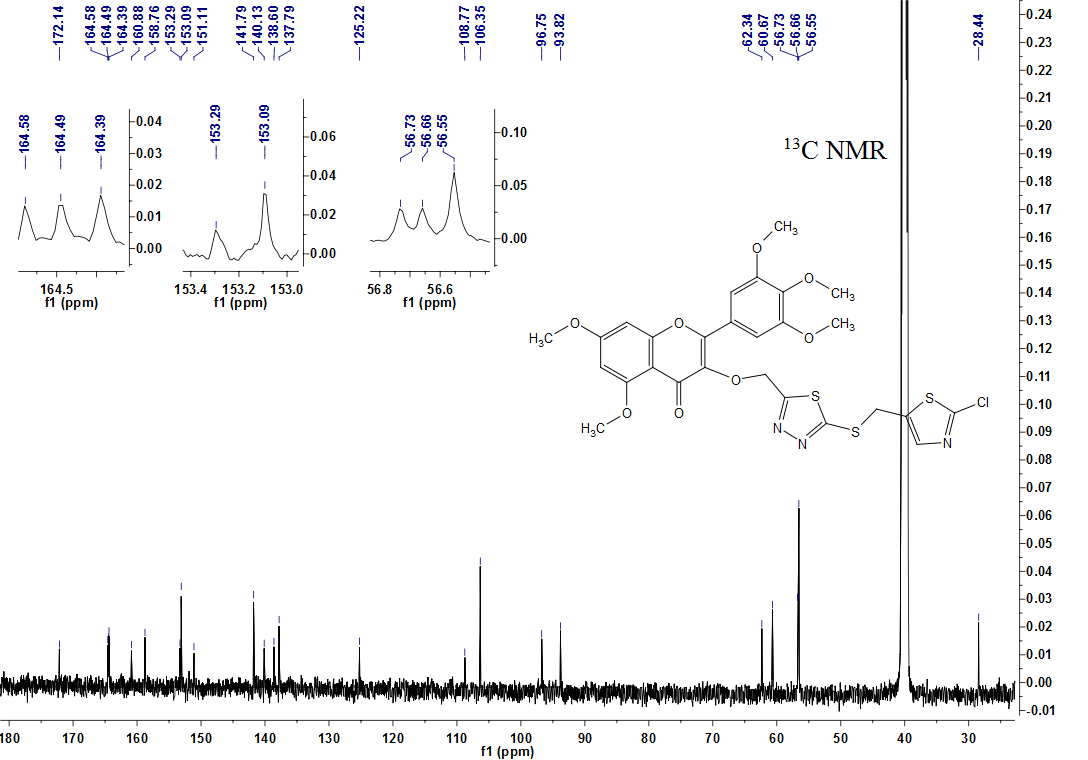
**

*Figure* ***S56****. HRMS spectrum of compound* ***3m***

Figure **S57**. IR spectrum of compound **3n**


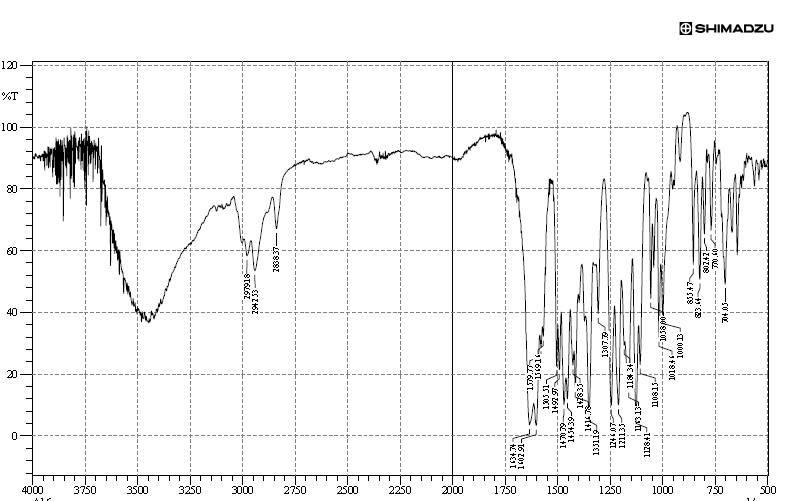


Figure **S58**. ^1^H NMR spectrum of compound **3n**

**
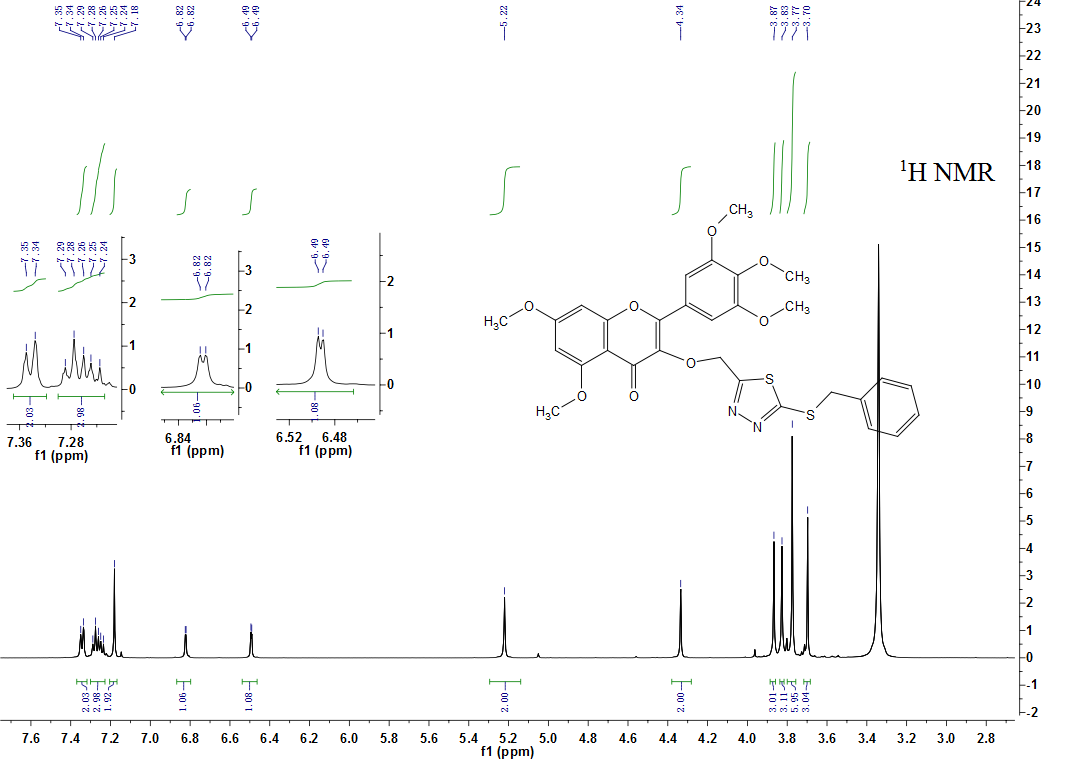
**

Figure **S59**. ^13^C NMR spectrum of compound **3n**

**
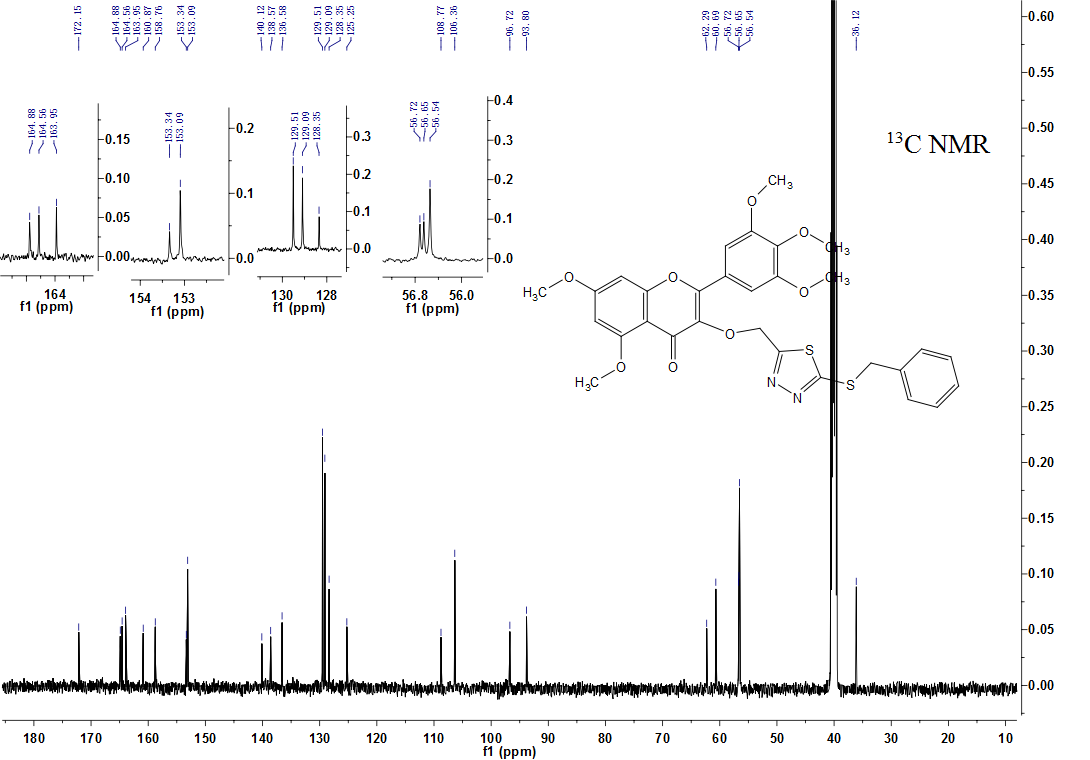
**

*Figure* ***S60****. HRMS spectrum of compound* ***3n***

Figure **S61**. IR spectrum of compound **3o**


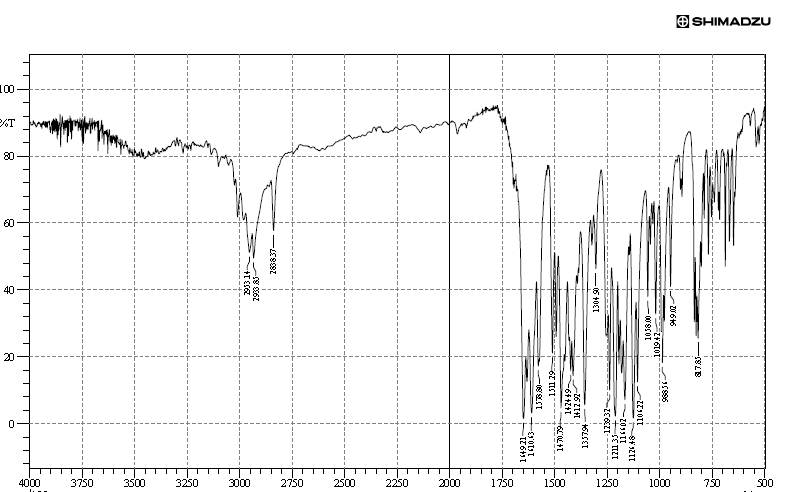


Figure **S62**. ^1^H NMR spectrum of compound **3o**

**
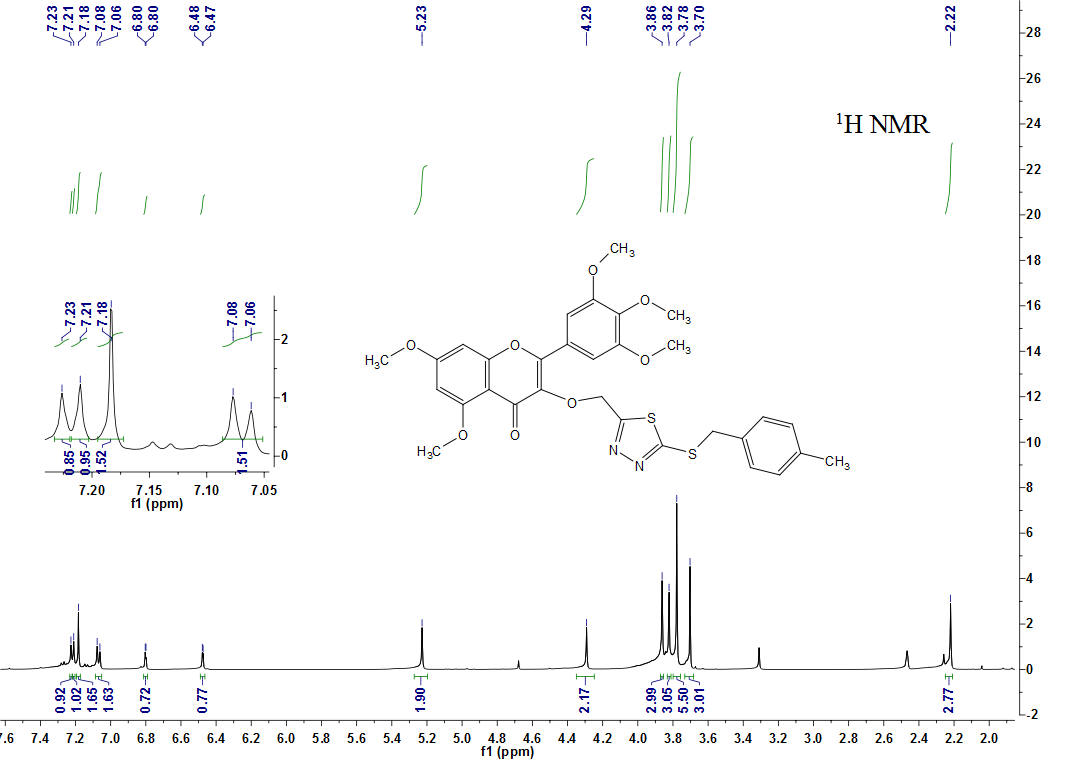
**

Figure **S63**. ^13^C NMR spectrum of compound **3o**

**
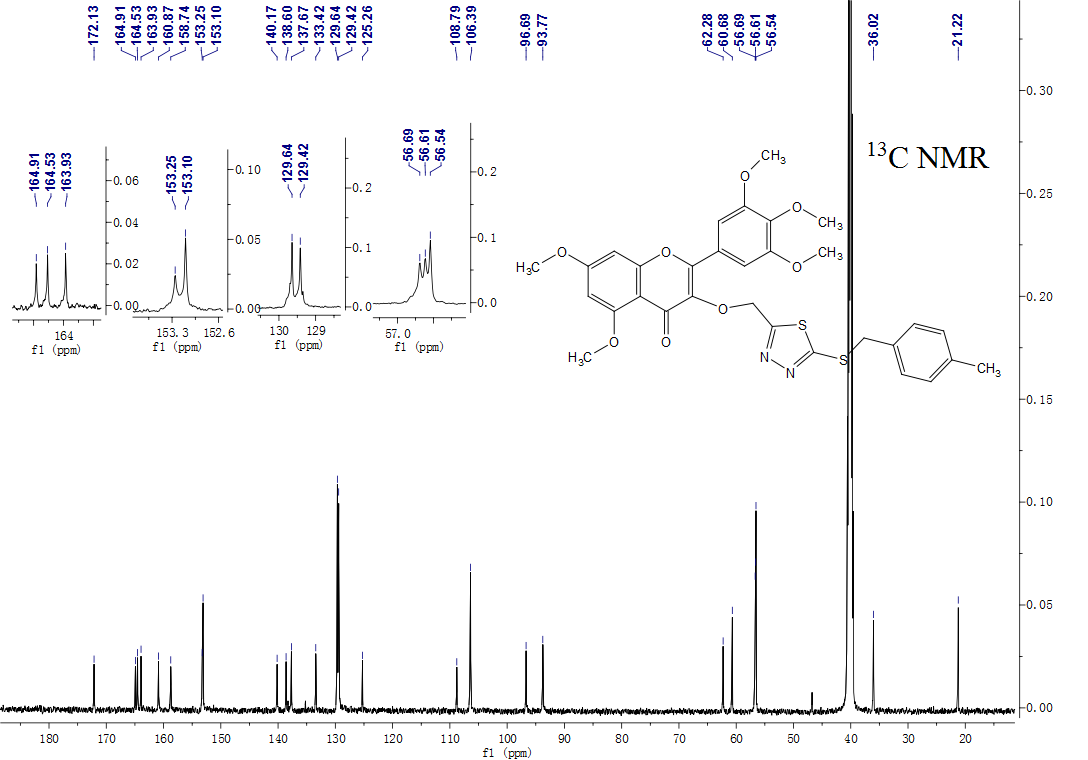
**

*Figure* ***S64****. HRMS spectrum of compound* ***3o***

Figure **S65**. IR spectrum of compound **3p**


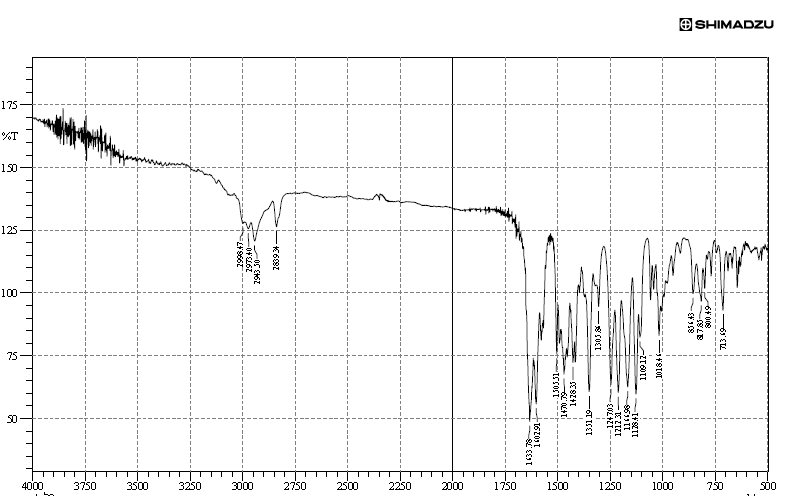


Figure **S66**. ^1^H NMR spectrum of compound **3p**

**
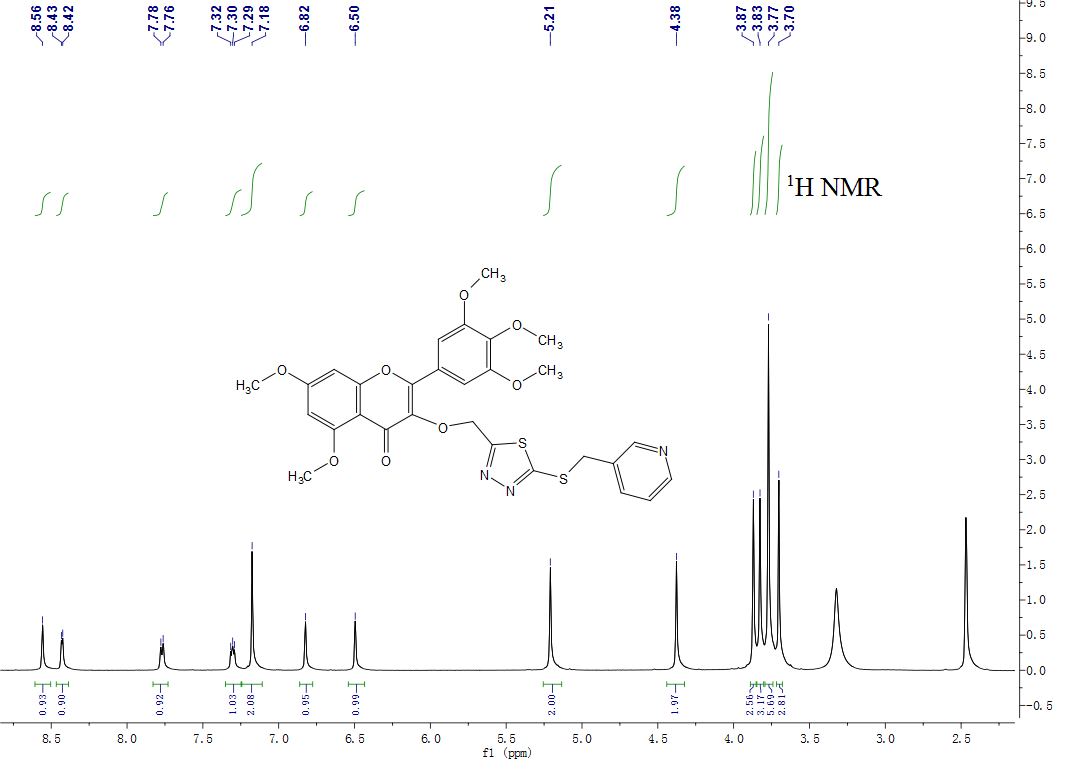
**

Figure **S67**. ^13^C NMR spectrum of compound **3p**

**
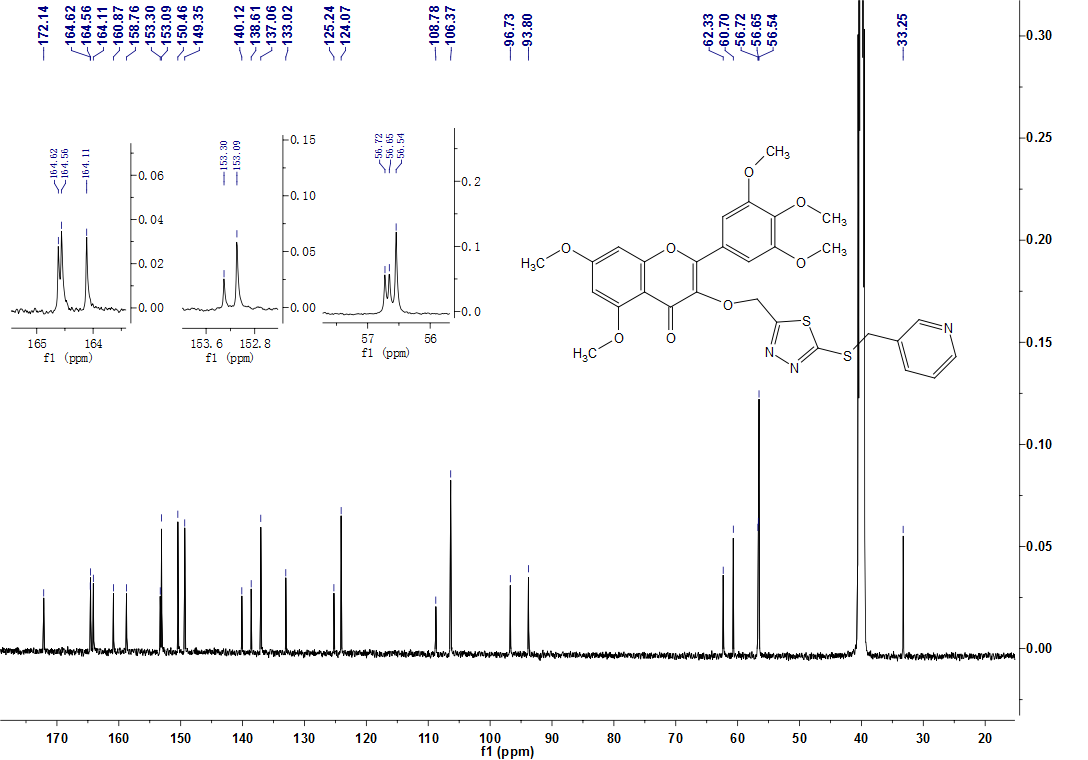
**

*Figure* ***S68****. HRMS spectrum of compound* ***3p***
